# The emergence of the visual word form: Longitudinal evolution of category-specific ventral visual areas during reading acquisition

**DOI:** 10.1371/journal.pbio.2004103

**Published:** 2018-03-06

**Authors:** Ghislaine Dehaene-Lambertz, Karla Monzalvo, Stanislas Dehaene

**Affiliations:** 1 INSERM, UMR992, CEA, NeuroSpin Center, University Paris Saclay, Gif-sur-Yvette, France; 2 College de France, Paris, France; Neurosciences Institute Stanford University, United States of America

## Abstract

How does education affect cortical organization? All literate adults possess a region specialized for letter strings, the visual word form area (VWFA), within the mosaic of ventral regions involved in processing other visual categories such as objects, places, faces, or body parts. Therefore, the acquisition of literacy may induce a reorientation of cortical maps towards letters at the expense of other categories such as faces. To test this cortical recycling hypothesis, we studied how the visual cortex of individual children changes during the first months of reading acquisition. Ten 6-year-old children were scanned longitudinally 6 or 7 times with functional magnetic resonance imaging (fMRI) before and throughout the first year of school. Subjects were exposed to a variety of pictures (words, numbers, tools, houses, faces, and bodies) while performing an unrelated target-detection task. Behavioral assessment indicated a sharp rise in grapheme–phoneme knowledge and reading speed in the first trimester of school. Concurrently, voxels specific to written words and digits emerged at the VWFA location. The responses to other categories remained largely stable, although right-hemispheric face-related activity increased in proportion to reading scores. Retrospective examination of the VWFA voxels prior to reading acquisition showed that reading encroaches on voxels that are initially weakly specialized for tools and close to but distinct from those responsive to faces. Remarkably, those voxels appear to keep their initial category selectivity while acquiring an additional and stronger responsivity to words. We propose a revised model of the neuronal recycling process in which new visual categories invade weakly specified cortex while leaving previously stabilized cortical responses unchanged.

## Author summary

Reading acquisition is a major landmark in child development. We examined how it changes the child’s brain. Ten young children were scanned repeatedly, once every 2 months, before, during, and after their first year of school. In the scanner, they watched images of faces, tools, bodies, houses, numbers, and letters while searching for a picture of “Waldo.” As soon as they started to acquire reading skills, a specific region of the visual cortex of the left hemisphere—called the visual word form area (VWFA)—started to selectively respond to written words. In every child, it was then possible to go backward in time and ask what this region was doing prior to reading. We found that written words invaded a sector of visual cortex that was initially weakly specialized, slightly responsive to pictures of tools, and that lay next to a face-selective region. Reading acquisition did not displace those initial responses but blocked their development, such that face-selective responses became stronger in the right hemisphere. Those results provide direct evidence for how education recycles the human brain by repurposing some visual regions towards the shapes of letters.

## Introduction

In both human and nonhuman primates, the ventral visual cortex comprises multiple specialized subregions that are involved in the visual recognition of image categories such as objects, faces, or places [1–5]. What is striking in humans, however, is that this mosaic of specialized regions is partially changed by the culture we live in. The acquisition of musical [6], mathematical [7,8], and reading abilities [9] leads to systematic changes in ventral visual organization.

In particular, in all adults who have learned to read—regardless of the writing system—a small region of the left ventral visual cortex within the depth of the left occipitotemporal cortex systematically activates in response to written words [9,10]. This region has been termed the visual word form area (VWFA). Although there is still debate about the exact function of the VWFA, which may vary according to a posterior-to-anterior gradient [11,12], it is widely accepted that the responsivity of this region is an excellent marker of reading acquisition [13]. For instance, a whole-brain comparison of brain activity evoked by letter strings in literate and illiterate adults isolates a specific site at the location of the VWFA, the activation of which is proportional to reading speed [14]. Similarly, the VWFA site appears when comparing functional magnetic resonance imaging (fMRI) images of children who have or have not learned to read, either as a group [15,16] or within the same individual [17–19]. In both children and adults, those changes are accompanied by a massive enhancement and left lateralization of the N170 component of event-related potentials evoked by written words [e.g. 17,20,21–23].

In the present work, we aimed to provide novel longitudinal data on the impact of the acquisition of reading on the representations of other visual categories in the ventral visual cortex. In adults, the VWFA systematically lands at a fixed location relative to a reproducible mosaic of regions partially specialized for objects, faces, bodies, and places [10,14, 24–26]. Little is known, however, about the development of this system in young children. In 9-year-old normal readers, the adult mesial-to-lateral gradient of preferred responses to houses, faces, and words is already present, with the expected left–right hemispheric asymmetries for words versus faces [15], but fMRI studies in younger children have underlined the protracted development of this mosaic of regions [27,28]. In particular, a selective response to faces is often difficult to isolate in the fusiform region in early childhood [27,28, but see 29] although a coarse mesial–lateral functional division at the level of the fusiform lobe can be suspected since infanthood [30]. The response to faces increases with age until late adolescence, while the activation for places and tools appears more stable along childhood [27,28,31,32]. A recent study indeed suggests that the face region is more plastic than the place region and continues to exhibit structural changes until adulthood [33].

In this context, it has been hypothesized that the acquisition of reading takes advantage of the preexisting organization and plasticity of this ventral visual cortex [34]. The theory of neuronal recycling proposes that cultural learning takes advantage of the prior organization of the cortex and repurposes some of its circuitry [35]. This could explain why expertise in reading primarily encroaches on the lateral sector of the ventral visual cortex, where late-childhood plasticity is maximal [33]. A combination of constraints, including preexisting connections to language areas [18,36], sensitivity to line junctions [37], and high-resolution representation of fovea shapes [26] would conspire to single out a specific cortical location as the most appropriate for the visual recognition of written words [38]. Reading acquisition would then displace the preexisting mosaic of visual categories in this region, leading to a reorganization that “makes space” for letter knowledge. In support of those ideas, the comparison of literate and illiterate adults [14,23] has revealed that the responses to written words overlap with those responding to objects, faces, and checkerboards and that as reading scores increase, face responses were slightly reduced in the left hemisphere and strongly increased in the right hemisphere. Similarly, in children, Monzalvo et al. [15] further showed that the right lateralization of the activation to faces increased with reading performance. Behavioral and event-related potentials have further supported the notion of a competition of words and faces for cortical space [23,39–41].

One problem, however, is that those studies relied on a comparison of distinct groups of subjects with variable ages and literacy scores. Such group comparisons are necessarily imprecise. Smoothing and intersubject averaging may lead to an apparent overlap between the cortical responses to different categories, even though those regions actually occupy well-delimited territories in individual subjects. Evidence on the development of reading within individual children is simply lacking. In the present study, our primary aim was therefore to obtain enough longitudinal data on a few individual children that they could be submitted to a single-subject analysis. To this aim, we scanned 10 children longitudinally at 6 different times, spread at approximately 2-month intervals before and throughout the first year of schooling (8 of them also came back for a seventh scan 1 year later). Furthermore, to better understand early cortical maps and their reorganization with reading, we presented the children with a broad array of age-appropriate pictures covering the 6 categories of letters, numbers, objects, faces, bodies, and places.

These longitudinal single-subject data depicting the evolution of ventral visual responses to words and other visual categories should allow us to clarify the topographical changes, dynamics, and cortical competition underlying reading development, and we aimed to clarify the following questions: how quickly does the VWFA emerge during reading acquisition? Does it immediately land at its usual adult location, or does it move during development? Do we observe a transient invasion of broader cortical territories followed by selective shrinking? Do other categories remain stable, or are they shifted away from the site that becomes specialized for letters? Can the VWFA site be predicted by a prior pattern of specialization for other categories, such as faces? Or, on the contrary, do word-specific voxels fall upon a sector that is initially poorly specified? And what is the relation between the development of face and word responses: are these categories competing for the same resources in a plastic region?

## Methods

### Ethics statement

This study is part of the project "Etude multimodale en neuropsychologie et imagerie du développement cérébral et cognitif et de ses relations avec la variabilité génétique," approved on June 16, 2011, by the ethical committee (CPP Kremlin Bicêtre, N° 11–008).

### Subjects

We received ethical permission to scan 10 healthy children at approximately 2-month intervals, from the end of kindergarten through the first year of school (July, September, November, January, March, and June; the school year started 15 September). An extension allowed us to rescan 8 of them for a seventh time at the end of the next year of school (June). The 10 children (5 boys and 5 girls) were selected from an initial sample of 14 children who were scanned at session 1 (July, kindergarten). The purpose of that first session was 2-fold: (1) for the children and their parents to realize what a scanning session was and (2) for us to see which children had trouble staying quiet in the scanner. We then selected the first 10 children who seemed quiet enough and agreed to come back throughout the year. All children and both their parents signed a written consent at the first session. Then, at each session, they were asked whether they agreed to continue.

**Fig 1:**
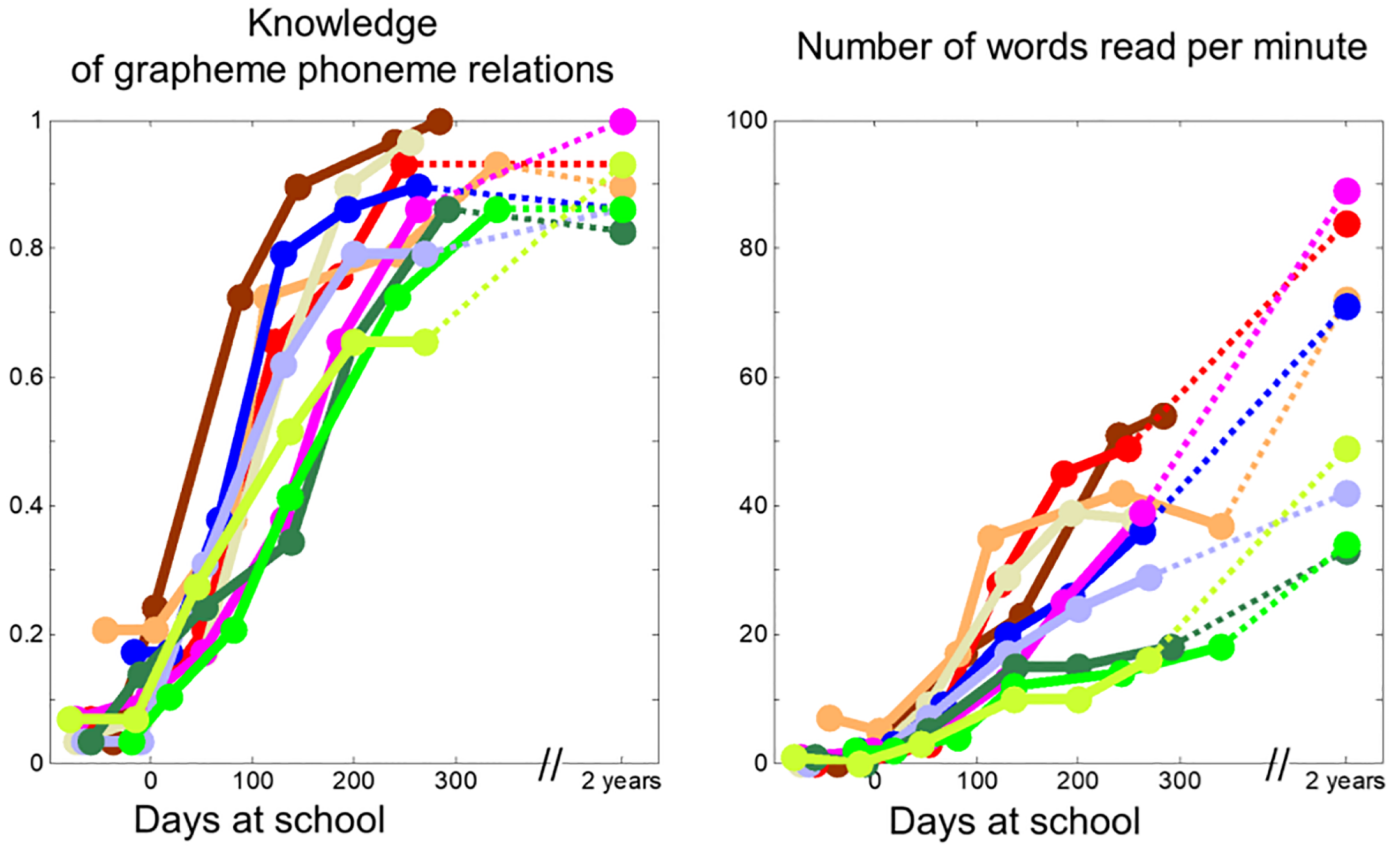
Evolution of reading performance as a function of schooling in each child across the 7 measurement times. Each color represents the performance of a given child. The norms for the number of words read per minute is 36.7 (±15.8) at the end of the second year of French school (LUM). S1 Data. LUM, “Lecture en une minute.”

At the first scan, children were aged 6 years 2 months on average (range 5:7 to 6:7). We ensured that they possessed little or no reading ability (number of words read in 1 minute = 0 to 7), thus probably selecting children in the lower half of the normal range. None had any known risk factor of reading impairment in their family history: their development was judged normal by their teacher and parents, and they exhibited normal-range performance in verbal and perceptual intelligence quotient (IQ) subtests (WISC IV) and Raven’s Colored Progressive Matrices. We assessed their reading level with 2 tests: “L’alouette,” a classic standardized French reading test, which consists of reading as fast and accurately as possible a meaningless text of 265 words within 3 minutes [42], and “Lecture en une minute” (LUM), a standardized list of words that children are asked to read as fast as possible for 1 minute [43]. At the end of the first year of schooling, their reading age (“alouette”) was, on average, +1 month (range −9 to +15 months) relative to their civil age, indicating normal development, and this value was −2.4 months (range −17 to +39) at the end of the second year (Fig 1). The number of correct words read in 1 minute (LUM) was 33.4 at the end of the first year (range 16 to 54) and 59.25 at the end of the second year (range 33 to 89), which is within or above the standardized norm at the end of the second year of school, i.e., 36.7 (±15.8) words per minute. We recently received news from 9 of the 10 children. All 9, now in their first year of secondary school (sixth grade), have followed a normal school curriculum without reading difficulties.

Just before each fMRI session, the reading level was assessed with a new list of words similar to the standardized LUM list. We also probed knowledge of the grapheme–phoneme code (BATELEM test) and other abilities affected by reading acquisition, including the following: rapid automatic naming of pictures (RAN), verbal short-term memory (forward and backward digit span; sentence span: correct repetition of sentences of increasing length), phonological awareness (EVALEC test, comprising deletion of the first syllable in 10 trisyllabic words, then of the first phoneme in 12 CVC words and in 12 CCV words [44]), vocabulary level (DEN48 test of picture naming [45]), number reading, and number dictation. Finally, to study the effect of reading acquisition on face processing, the face recognition test from NEPSY [46] was evaluated. After having memorized 16 children’s faces, the child had to point to the studied face among 3 faces, across 16 trials. This test was done immediately after the encoding phase and after a 30-minute delay. Except on the first, sixth, and seventh sessions (1 year apart)—for which we used standardized tests—in the intermediate sessions, we constructed equivalent stimulus materials in order to avoid test–retest effects as much as possible.

### Stimuli

**Fig 2:**
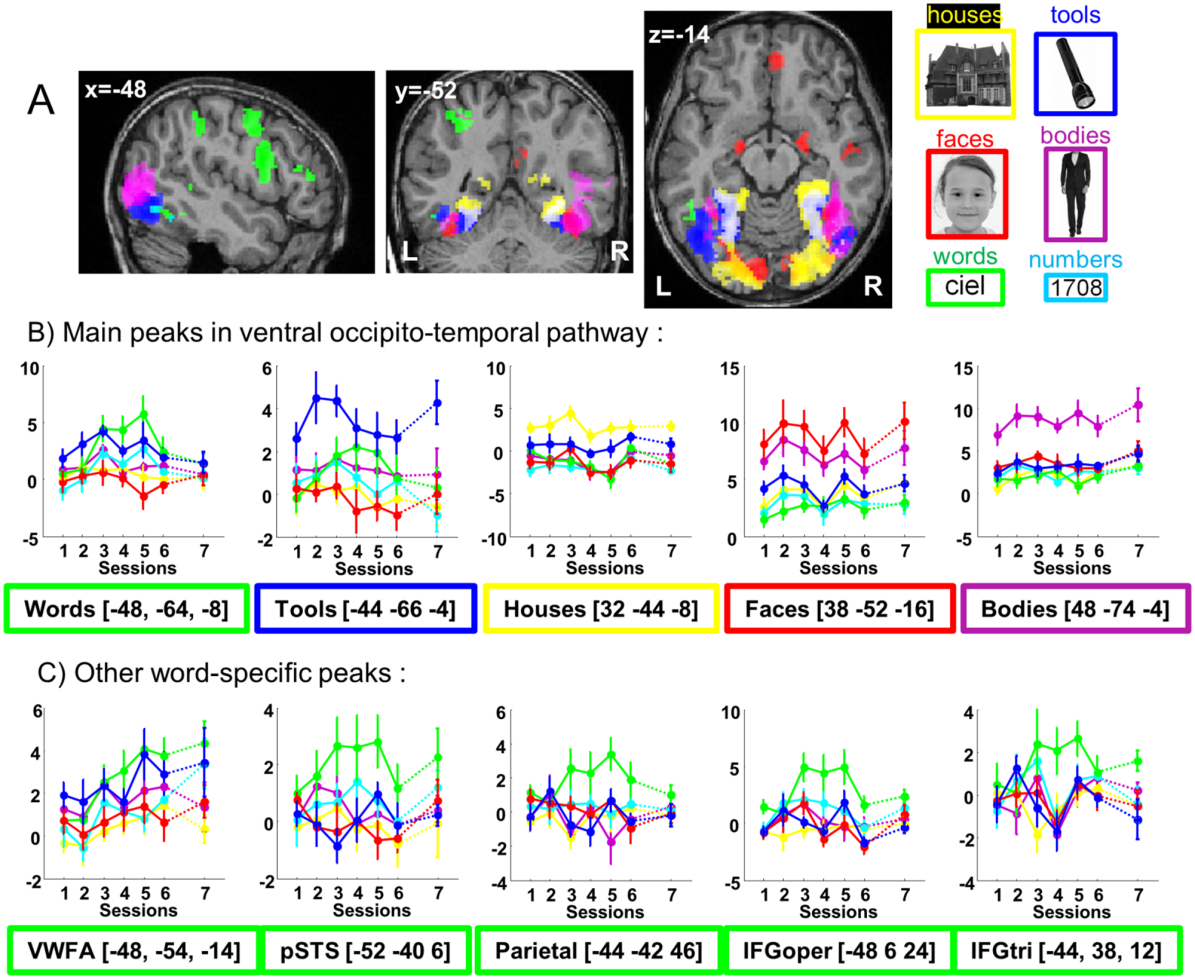
Category-specific activations across all 7 sessions and 10 children. (A) Each category is contrasted relative to the other pictures (omitting the grids) while pooling across all 7 sessions. On the right, one example of each presented visual category. The color of the frame indicates the visual category of the specific activation on the slices and of the lines in the plots. The significant clusters (*p* < 0.001 corrected at the cluster level at *p* < .05) are presented over an individual child brain. No significant cluster was observed for numbers. See S2 Fig to see the activation for each category separately and S3 Fig for an example in an individual child (child 2). (B) Plots show the evolution over sessions of the betas for each category, at the primary peak in ventral visual cortex for each category and (C) at the peaks of the word specific activations in the whole brain. The first 6 sessions were evenly spaced during the first year of school, while the seventh session occurred at the end of the second year. Note that while the contrast used to create this image weighted all 7 sessions equally, plots revealed linear (e.g., VWFA) as well as quadratic (e.g., parietal, IFG) profiles, which are further analyzed in the text. (https://neurovault.org/collections/3457/). IFGoper, inferior frontal gyrus opercularis; IFGtri, inferior frontal gyrus triangularis; pSTS, posterior superior temporal sulcus; VWFA, visual word form area.

For the fMRI paradigm, subjects were presented with stimuli belonging to the categories of houses, objects, faces, bodies, words, and numbers (see Fig 2 and S2 Fig). Two additional categories of high-frequency and low-frequency grids with variable orientations were also presented. Each category comprised 60 different exemplars, either using black and white pictures (for houses, objects, faces, and bodies) or 4-character strings (for letters and numbers). The words were frequent, regular words encountered by young readers, as specified in Manulex, a lexical database compiling the frequency of occurrence of words in 54 scholarly French reading books [47]. Faces were front views of male and female children’s faces. Bodies were standing male and female adult bodies; to avoid face responses, their head fell outside the picture frame (see an example in Fig 2). Objects were pictures of objects frequently encountered in a child’s daily life (scissors, spoons, shoes, etc.). Six subsets comprising 10 exemplars of each category were created, to be successively used in the 6 scanning sessions. The order of the subsets was different for each child.

### Functional imaging paradigm

We used a miniblock design. Blocks of 6 images belonging to the same category were randomly selected and presented during 1 second each, thus forming a 6-second block. Blocks were separated by a variable interblock interval of 2.4, 3.6, or 4.8 s (mean of 3.6 seconds). Thus, a new series of images was presented on average every 9.6 seconds. The order of the categories was randomly chosen, with the constraint that each category was presented 3 times in a functional run (8 categories × 3 repetitions = 24 blocks of 6 images each).

As in our previous work [14,15], we used an easy incidental target-detection task, the sole purpose of which was to maintain attention in all miniblocks, independently of reading or schooling level. Within each block, a target (the picture of the cartoon character Waldo) had a 33% chance to appear, replacing 1 of the 6 images (excluding the first 2 images of the block). Therefore, an average of 8 targets appeared during a run of 24 blocks. Children were instructed to press a button as soon as they detected Waldo. This task was used to keep the child’s attention focused toward the visual stimuli. The total run duration was 3’ 58”. In each fMRI session, 4 runs were acquired, except for the first and sixth sessions, in which only 3 runs were acquired due to the additional tests and sequences proposed to the children in these sessions. In the seventh session, 4 runs were acquired except for 2 children who asked to stop the acquisition after the third run.

### Experimental procedure

After training to remain still in a mock scanner (only for the first session), children were brought to the 3T MRI scanner (Siemens Trio). They were protected with noise-protection earphones, and a mirror system above their head allowed them to see the visual stimuli presented on a screen at the end of the tunnel. The images were viewed from a distance of 120 cm with an approximate view angle of 6 degrees. Stimulus presentation and behavioral responses collection were performed using PsychToolbox (http://psychtoolbox.org), a free Matlab toolbox (MathWorks, Natick, MA, US). In each session, T1 images were first acquired (voxel size = 1 × 1 × 1 mm), then functional images were collected (100 EPI volumes with TR = 2.4 seconds, TE = 30 millisecond matrix 64 × 64 × 40, voxel size = 3 × 3 × 3 mm in each run). At the first, sixth, and seventh sessions, this sequence was completed by diffusion tensor imaging (DTI) and a second fMRI sequence studying audiovisual representations, which are not reported in the current paper. To reduce head motion, the quality of the MRI images was checked after each sequence acquisition and functional run, and verbal feedback was given to the child.

### Preprocessing

To correct for motion within each run, images were first realigned using the corresponding tool provided by SPM8 (http://www.fil.ion.ucl.ac.uk/spm/), including both estimation and reslicing steps. The target image was the mean of all images, except if movement during acquisition corrupted the mean image. In that case, the first image was used. S1 Fig shows the average amount of detected movement, computed as the maximum absolute value of the three translation and three rotation parameters provided by SPM. Average movement amounted to a few millimeters in translation and a fraction of a degree in rotation, often due to a sudden movement (e.g., cough), whereas the child was quiet most of the time. Each fMRI volume was then visually inspected one by one. Using the Matlab toolbox ArtRepair (http://cibsr.stanford.edu/tools/human-brain-project/artrepair-software.html), images affected by excessive intravolume movement artifacts (stripes, severe shape or size distortion) were replaced by linear interpolation of previous and subsequent images or by nearest-neighbor interpolation when the damaged volume was the first or the last or when several consecutive images were affected [48]. The percentage of rejected images remained low, with the majority of sessions requiring no correction at all (44/68 fMRI runs) or less than 4 images (61/68 = 90%). The mean rejection percentage across the 68 sessions was 0.77% (standard deviation = 1.1%; range 0%–16%).

Corrected images were then ready to undergo the rest of the preprocessing, i.e., slice timing, coregistration to the anatomy acquired on the same session, and normalization. For normalization, the T1-weighted anatomical images were first normalized to the standard European adult MNI template. This step segmented the images automatically into different tissue classes (grey matter, white matter, and nonbrain, i.e., cerebrospinal fluid and skull) using the “New Segmentation” option in SPM8. By averaging those segmented images across all 10 subjects and all 7 sessions, 3 tissue probability maps were obtained. The original T1 images were then normalized a second time, this time using as a target template those average images arising from our own cohort of children. The highly accurate alignment of the 6 or 7 anatomies obtained from the same child was visually verified using the CheckReg tool in SPM8. Finally, the normalization matrix was applied to all EPI images of the corresponding session, with a final resampled voxel size of 2 × 2 × 2 mm.

### Statistical analyses

#### Behavioral results

To capture changes related to school and reading acquisition, we entered the behavioral performance collected at each session in multiple regression analyses with 2 main independent variables: time spent at school (i.e., the number of days between the test date and the entrance at school, 15 September) and reading speed (i.e., the number of correct words read in 1 minute estimated by LUM).

#### Single-subject fMRI model

In each subject, the unsmoothed EPI images were modelled across sessions using the canonical SPM hemodynamic response function and its time derivative convolved with the 8 experimental conditions (low- and high-frequency grids, houses, tools, bodies, faces, words, and numbers). The 6 movement parameters were entered as regressors of noninterest. Because subjects occasionally shifted head position across runs, some voxels at the border of the brain failed to be properly acquired in some fMRI volumes; the SPM masking parameter was therefore set to keep all voxels with at least 80% of valid data across all fMRI sessions. Contrasts were weighted according to the number of runs in each session.

#### Group fMRI model

Unsmoothed contrasts for each category relative to rest and from each session were entered in a second-level analysis across the 10 subjects. An analysis-of-variance model included category and sessions as within-subject factors. We also implemented a regression model with category, age, and the number of words read in 1 minute (LUM) as regressors. We report significant results when voxels were significant at *p* < 0.001 and formed a contiguous cluster the extent of which was significant at *p* < 0.05 (family-wise error [FWE]), corrected for multiple comparisons across the set of analyzed voxels (denoted as p_cor_). For most of our analyses, this set corresponded to the whole brain (i.e., a mask image of the voxels for which at least 50% of the subjects had data). Results were visualized using xjView toolbox (http://www.alivelearn.net/xjview). As explained in the Results section, grids induced a larger activation than other categories not only in calcarine cortex but also in broad areas of dorsal visual pathway. This was probably due to perceived movement induced by the constant change in line orientation between the images within a block, thus making it a poor control for most analyses. We therefore did not consider them further in the analyses except when explicitly written. To study the organization of category specificity, we contrasted each category versus all other categories except grids. When the effects of sessions, reading level, or days at school were considered, we first examined the effect of these factors on each category versus rest; then, we investigated their category specificity by contrasting each category to all other categories except grids (for instance, words minus [tools, bodies, faces, houses, and numbers]).

#### Individual analyses within ventral visual cortex

To further understand the influence of reading on ventral visual cortex, we restricted the analyses to an anatomical ventral occipitotemporal region delimited by a box with the following MNI coordinates: −70 ≤ x ≤ −20; −82 ≤ y ≤ −33; −28 ≤ z ≤ +4 (called hereafter “ventral mask”). These limits were determined such that they systematically encompassed the occipitotemporal reading-related (RR) voxels in all subjects. Then, in order to obtain individual masks, we intersected this box with the subject-specific mask for grey matter and excluded cerebellar voxels thanks to a cerebellum template (“SUIT,” from reference [49]). On average, this ventral mask comprised 4,510 voxels, i.e., 36,080 mm^3^ (range 3,933–4,899 voxels). Its symmetric in the right hemisphere comprised 4,487 voxels, i.e., 35,896 mm^3^ (range 4,067–4,907 voxels) with no significant left–right difference (t[1,9] < 1).

In each subject, we recovered the voxels that were more responsive to one category than to others (voxel *p* < 0.001) within the left and right ventral masks for each session (1 to 6). We analyzed the following variables: (i) the number of significant voxels (*p* < .001) for each category and session, (ii) the distance in the left hemisphere of the barycenter of each specific category activation from the VWFA barycenter, and (iii) the distance of each category activation barycenter from the FFA barycenter (separately in the left and right hemisphere). The WVFA (and FFA) barycenter was determined as the mean x, y, and z of the significant voxels (*p* <. 001) in the contrast [words > others] and [faces > others] in sessions 6 and 7 in each child. The barycenter of the significant activation (*p* < .001) for each specific category was retrieved for each session and child, and its Euclidian distance with the WVFA (and FFA) barycenter was computed and entered in separate ANOVAs with category excluding words (or faces) (i.e., 5 levels in each case) and sessions (1–6 sessions) as within-subject factors.

In a second series of analyses, we aimed to study how voxels become (or are) category specific, in particular how they become word specific during reading acquisition. Therefore, we divided the previous individual masks in RR and non–reading-related (nRR) voxels. RR comprised all voxels significant at *p* < 0.001 in the contrast [words > others] in sessions 6 and 7, whereas nRR corresponded to all the other voxels of the child’s personal ventral mask. We did not apply a cluster-extent threshold, in order to identify even isolated voxels. The mean activation across all voxels of the RR versus nRR voxels were compared for each of the first 5 sessions using paired *t* tests to determine when a word-specific response appeared. For these analyses, session 6 cannot be considered because this session, with session 7, is used to construct RR and nRR regions of interest (ROIs).

We also analyzed whether an early preference for a particular category was observed in the RR voxels. The average activity evoked by each of the 4 nonsymbolic image categories (tools, houses, faces, and bodies) relative to rest, in each of the 5 initial sessions, was submitted to an ANOVA with categories (4 levels) and sessions as within-subject factors. We extended these analyses to the other category-specific ROIs. In each case, ROIs were defined in each child using the contrast [category X > others] (*p* < .001) in sessions 6 and 7, and analyses were performed on independent sessions (1 to 5).

Conversely, we also studied the evolution of voxels already category-specific in sessions 1 and 2. We did the same analyses as above but defined the category- and noncategory-specific voxels in the first 2 sessions for each of the 4 nonsymbolic image categories (tools, houses, faces, and bodies) relative to rest and analyzed sessions 3 to 6.

#### Multivariate patterns of category selectivity

We extracted the pattern of activation for each category relative to rest over the whole set of gray matter voxels of the ventral mask and probed how reproducible this pattern was from one session to the next (i.e., over a delay of approximately 2 months). We quantified this reproducibility by computing the correlation coefficient r between the vector of activation for one category relative to rest, at sessions *n* and *n* + 1, and comparing this correlation coefficient within category (e.g., correlation between the vectors of activity for faces in the two sessions) versus across categories (e.g., correlation between the vector for faces at session 1 and for houses at session 2). For reference, S8 Fig shows the full matrix of correlation coefficients between the patterns evoked by each of the 6 categories (relative to others) in each of the 7 sessions. It indicates a great degree of reliability across sessions. The individual correlation coefficients were entered in an ANOVA with factors of time (5 levels from sessions 1–2 to 5–6), category (6 levels), hemisphere (2 levels), and correlation type (within- versus between-category). We replicated this analysis while restricting the activation vectors to the VWFA voxels and, successively, to each of the category-specific ROIs defined in sessions 6 and 7.

## Results

### Behavior

All behavioral variables assessing reading—which were collected on each fMRI session during the first year of school—except RAN and vocabulary showed a significant increase with days-at-school: reading speed (r^2^ = 64%, *p* < 10^−13^), knowledge of grapheme−phoneme relations (r^2^ = 85%, *p* < 10^−25^), metaphonological performances (r^2^ = 42%, *p* < 10^−7^), backward digit span (r^2^ = 18%, p = .0004), word span (r^2^ = 26%, *p <* 10^−4^), number and digit reading (respectively, r^2^ = 14%, *p* = 0.003 and 22%, *p =* 0.0001), and number and digit dictation (respectively, r^2^ = 15%, *p =* 0.002 and 28%, *p <* 10^−4^). Time spent at school affects forward digit span only when the seventh session was included (r^2^ = 36%, *p <* 10^−7^). Fig 1 illustrates this relationship for 2 key variables: knowledge of grapheme−phoneme relations and reading speed (number of words read in 1 minute). Both were low and stable for the first 2 sessions before school, then sharply increased. At the end of first grade, most grapheme-knowledge relationships were mastered by all children, but reading speed remained highly variable.

Two regression models were used to attempt to characterize this variability. First, a multiple regression with both days-at-school and age indicated, unsurprisingly, an effect of the former but not the latter (respectively, *p <* 10^−5^ and *p =* 0.14, r^2^ = 65% for 6 sessions; and *p <* 0.001 and *p =* 0.24, r^2^ = 69% when 7 sessions were considered). Second, another multiple regression examined whether this effect was mediated by the evolution of 4 key cognitive variables: knowledge of grapheme−phoneme relations, metaphonological capacities, RAN, and effortful short-term memory (backward digit span). The results indicated that days-at-school was no longer significant and that knowledge of grapheme−phoneme relations and RAN mostly contributed to reading speed during the first year of school (respectively, *p <* 10^−6^ and *p =* 0.03, r^2^ = 81%). Because reading speed thus appears to summarize many aspects of the evolution of reading ability both within and across subjects, this variable was selected as the main regressor to study the evolution of brain activity as in our previous work in adults and dyslexic children [14,15].

Face memory ability also increased with days-at-school during the same period of 2 years (r^2^ = 26%, *p <* .10^−4^). The same regressions as above indicated that (1) days-at-school was a better predictor when pitted against age (respectively, *p =* 0.04 and *p =* 0.70, r^2^ = 24%); and (2) none of the above 4 cognitive variables were better predictors than days-at-school although backward digit span approached significance (*p =* .07); and (3) the same conclusion was reached when reading speed was pitted against days-at-school (respectively, *p =* 0.88 and *p =* 0.02; r^2^ = 24%). Those results suggest that face recognition may be influenced by schooling yet independently of reading.

During fMRI, only a minimal detection task (detection of a specific picture of a cartoon character Waldo) was required. There were no misses, and the mean reaction time (RT) to the target was 978 milliseconds (882 to 1,004 milliseconds across subjects and 933 to 1,001 milliseconds across sessions). The mean RT was stable across sessions (F[6,52] = 1.7; *p* > 0.1).

### fMRI group analysis

We first examined category-specific activations when pooling across all 7 sessions (Fig 2 and S2 Fig for glass-brain views of the category-specific activations). When compared to the other categories, grids induced larger activation mainly in the calcarine scissures and areas of the dorsal occipitoparietal pathway, probably due to perceived movement induced by the constant change in line orientation (S2A Fig) across the successive images of the block. Because this category was so different from the others, we did not consider this condition further and only compared each category to the mean of the other “pictures” categories (i.e., tools, words, numbers, faces, bodies, and houses). For these visual categories, a mosaic of specific responses to each category was observed in ventral extrastriate areas, similar to that reported in adults. From medial to lateral, the preference shifted from houses to bodies and objects (Fig 2 and S2 and S3 Figs). Tools activated 2 separated ventral foci as described by Hasson et al. [50] in adults. Faces mainly activated both fusiform gyri and amygdala, plus the right superior temporal sulcus. Interestingly, bodies induced a much larger response than faces in the inferior temporal and posterior middle temporal regions, while the converse effect was seen in the medial occipital region (S2 Fig). Numbers did not elicit any larger response relative to the other visual categories. Words, however, elicited an extended pattern of activation, with significant clusters observed not only in the VWFA but also in posterior temporal sulcus, parietal, and inferior frontal regions, all in the left hemisphere (Fig 2).

We also pitted our two symbolic categories—letters and numbers—against each other (S2B Fig). Relative to numbers, words elicited stronger activations in the left frontal (240 vox, p_cor_ < 0.001, z = 5.11 at [−52 10 24]), left parietal (115 vox, p_cor_ < 0.001, z = 4.64 at [−32 −52 44]), and left fusiform regions (56 vox, p_cor_ < 0.001, z = 4.06 at [−40 −56 −8] and z = 4.00 at [−48 −55 −8]), confirming the presence of an early specialization for letters over numbers at the left VWFA site even at this early age. No significant cluster was observed for the reverse comparison (numbers > words).

**Fig 3:**
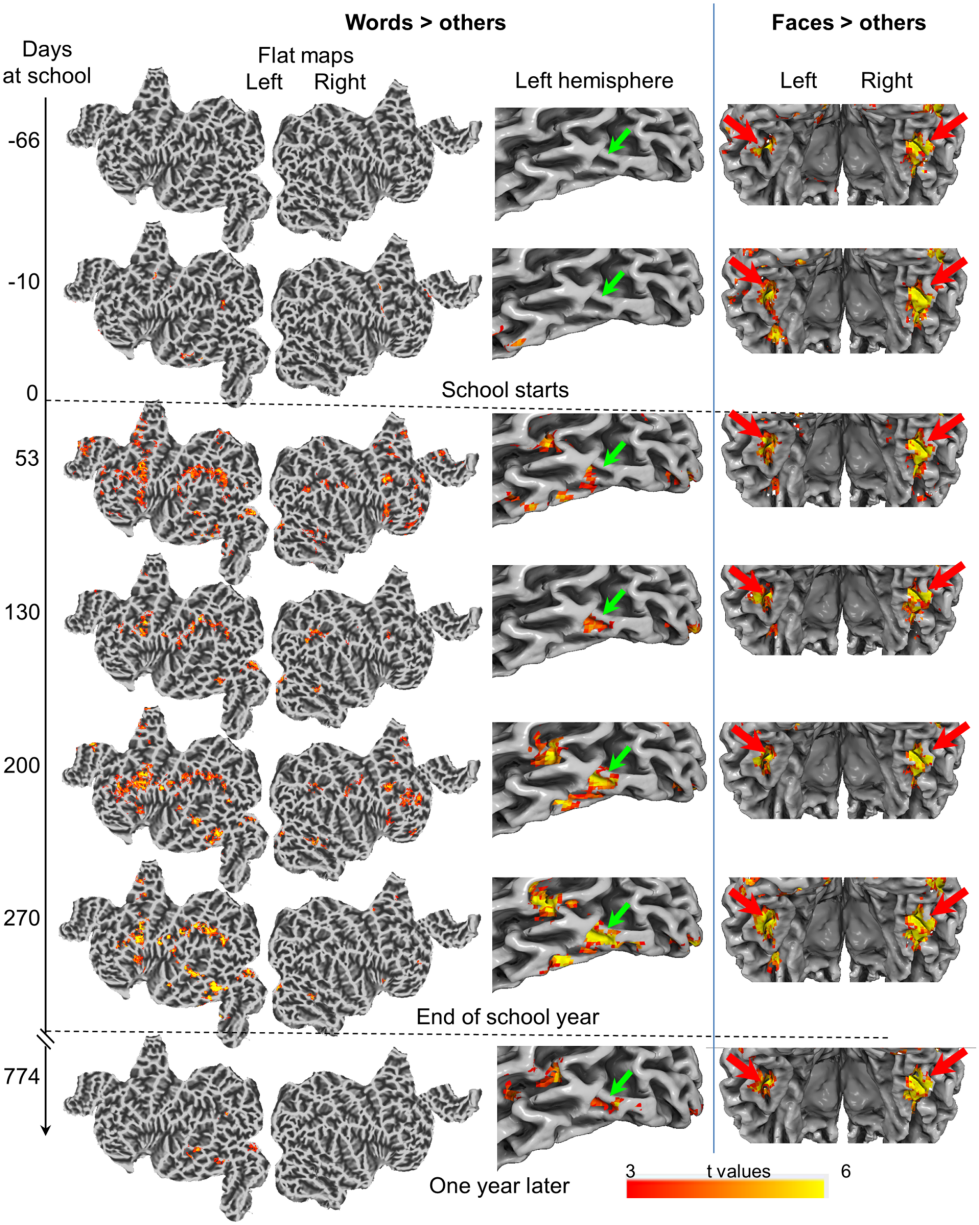
Evolution of activation in a child (child 2) who was scanned 7 times (top to bottom). The left columns show the evolution of words > others on whole-brain flat maps and on a slightly inflated view of the left hemisphere. There was no activation of the RR circuit in the first 2 sessions, prior to school onset. Once schooling started, a VWFA activation was quickly detected, increasing in intensity with time and remaining detectable 1 year later. Parietal and prefrontal areas were transiently activated. In the middle column, zoom on the left ventral visual region. The green arrow indicates the VWFA. The right-hand column, ventral areas viewed from the back, illustrates the stability of face-related activations (contrast = faces > others, red arrows). See S3 Fig for the other categories. All contrasts at voxelwise *p <* 0.001 and clusterwise *p <* 0.05 FWE corrected. FWE, family-wise error; RR, reading-related; VWFA, visual word form area.

Second, we examined the changes in those activations across the 7 sessions, spread over the first year of reading acquisition, with an additional measurement at the end of the second year of schooling. Fig 3 shows an example of the evolution of the activation to words and to faces in an individual child (see S3 Fig for the other visual categories). In the group analysis, there was a linear increase with sessions of the activation to words relative to rest in several areas of the left hemisphere: in the anterior part of the fusiform region (VWFA), the occipital area, the posterior superior temporal sulcus, the precentral region, and both inferior parietal areas (see coordinates and Z scores in Table 1). A similar increase with sessions was seen for the activation to numbers relative to rest in the right inferior parietal region (77 vox, p_cor_ < 0.001, z = 4.14 at [28 −60 36]) and for houses at [32 −78 32] (z = 4.16, 56 vox, p_cor_ = 0.002). No significant linear effect was observed for the other categories. No area showed a significantly larger increase to one category than to the others. However, when compared to grids, a specific increase was seen for words in the VWFA (74 vox, p_cor_ < 0.001, z = 4.72 at [−50 −58 −14]). Session-by-session comparisons (Fig 2) indicated that the difference between words and other categories was absent from sessions 1 and 2 (before or at the onset of schooling) and first became significant in session 3 for prefrontal and temporal activations or session 4 for the VWFA, i.e., about 2 or 4 months, respectively, after school onset.

**Table 1:** Linear increase of the word activation relative to rest across the 7 sessions in all children.

Region	MNI coordinates x y z	Z score	Cluster size	*P*_FWEcor_
Left inferior parietal	−32 −48 34	5.12	89	<0.001
Left fusiform	−42 −50 −22	5.08	160	<0.001
Left inferior occipital	−32 −92 −10	5.04	116	<0.001
Left superior temp. sulcus	−54 −30 0	4.85	32	0.05
Left precentral	−36 0 34	4.62	32	0.05
Left superior occipital	−24 −62 34	4.61	149	<0.001
Right inferior parietal	30 −62 36	4.13	47	0.006

Abbreviations: MNI, Montreal Neurological Institute; *P*_FWEcor_, p family-wise error–corrected.

**Fig 4:**
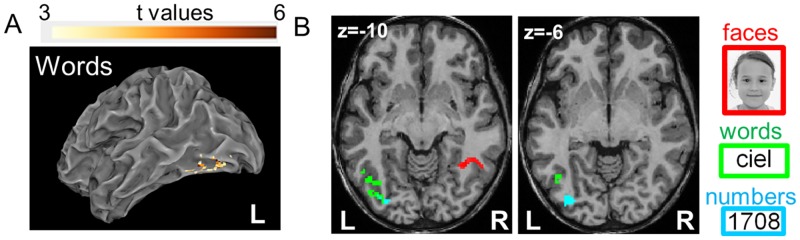
Brain activations correlating with reading speed as it evolves across scanning sessions, independently of age. (A) Effect of reading speed (LUM) on word-evoked activations relative to rest, in a model in which age was also entered as a covariable. (B) Reading speed effect on the activation evoked by faces, words, and numbers. Activation to words (green) and numbers (blue) increased with reading speed in the left occipitotemporal pathway. Reading expertise was also associated with increased responses to faces in the right fusiform gyrus (red). No significant effect was seen for the other categories. All contrasts at voxelwise *p <* 0.001 and clusterwise *p <* 0.05 FWE corrected. FWE, family-wise error; LUM, “Lecture en une minute.”

We next used regressions to evaluate whether behavioral measures of reading would predict the evolution of brain activity across sessions, independently of age (see Methods and Fig 4). A significant linear effect of reading speed (variable LUM = number of words read in 1 minute) was found on activation to words relative to rest in left occipitotemporal cortex, at around the VWFA site (75 vox, p_cor_ < 0.001, z = 4.66 at [−42 −66 −16]), at a more posterior occipital area (86 vox, p_cor_ < 0.001, z = 4.72 at [−38 −80 −10]), and in the right cerebellum (101 vox, p_cor_ < 0.001, z = 4.26 at [20 −50 −30]). This correlation was stronger for words than for other categories in left occipital (52 vox, p_cor_ = 0.003, z = 4.58 at [−42 −78 −12]) and occipitotemporal areas (110 vox, p_cor_ < 0.001, z = 5.25 at [−44 −64 −8]), indicating that these activations were reading specific.

In addition, interestingly, activation to categories other than words also correlated with reading speed. For faces relative to rest, an increase of activation with reading speed was found in the right-hemispheric fusiform gyrus, at or near the FFA site (48 vox, p_cor_ = 0.005, z = 6.08 at [38 −52 −10]). Furthermore, this increase remained significant when contrasting faces versus other categories (78 vox, p_cor_ < 0.001, z = 6.67 at [38 −52 −12]). This finding confirms the prior finding in adults (literate versus illiterate [14]) and in children (normal readers versus dyslexic [15]) that reading acquisition correlates with a right-hemispheric shift of face responses. For numbers relative to rest, an increase with reading speed was found in a left occipital area (46 vox, p_cor_ = 0.01, z = 5.00 at [−36 −82 −6]), but no difference with other categories was seen. For other categories, there was no significant correlation. Also, in those regressions, no specific increase in activation was found with age or with the number of days at school, for any category relative to all others.

The temporal profiles of activation to words presented in Fig 2 (bottom row) suggested that, in addition to monotonic increases, there might also be transient activations in the first months of reading acquisition that later reduced or vanished. To evaluate this possibility, we examined a quadratic contrast across the 7 sessions for word activations. The quadratic effect was significant in several left-hemispheric clusters: inferior frontal gyrus pars opercularis (p_cor_ = .043, z = 4.53, 33 vox at [−52 8 24]), anterior cingulate (36 vox, p_cor_ = .026, z = 4.33 at [−6 12 48]), anterior occipitotemporal sulcus (42 vox, p_cor_ = .012, z = 4.27 at [−48 −46 −8]), and anterior insula (34 vox, p_cor_ = .037, z = 4.18 at [−30 18 8]). Left inferior parietal cortex was also present at a less stringent false discovery rate (FDR)–corrected threshold (26 vox, p_FDRcor_ = .025, p_FWEcor_ = .13, z = 4.50 at [−26 −66 32]). Only the left parietal cluster remained significant when compared to the other visual categories or to the grids.

Overall, the group analysis confirmed that ventral occipitotemporal organization in the present group of 6-year-old children was similar to previous studies of adults. Reading acquisition primarily impacted word and number activations in the left occipitotemporal region. Face- and body-selective responses were seen at the earliest age, and face selectivity increased with reading speed in the right hemisphere. To better understand how these responses were organized, we next examined the voxel-specific evolution of category specificity in each subject.

### Properties of the VWFA and other category-specific areas in individual subjects

We asked whether the VWFA could be identified in each individual subject using the contrast for activation to words relative to other visual categories, once reading was established (we used sessions 6 and 7 or—in the 2 subjects who could not return for session 7—session 6 only). At our standard threshold (*p <* 0.001, cluster–FWE-corrected *p <* 0.05), 8 out of 10 subjects showed a significant cluster at, or near, the classical VWFA location in the left occipitotemporal area (see S4 and S5 Figs for individual brain maps and S1 Table for coordinates). Concerning the last 2 children, 1 had a cluster at the correct location but not significant when FWE-corrected (z = 5.67 at [−50 −62 −16], 40 vox, p_cor_ = .092, *p =* .035 FDRcor), and the other, who was the second worst reader, showed only a significant occipital activation for the same contrast (83 vox, p _cor_ <.001, z = 5.88 at [−16 −94 −10]) and a very small peak at the VWFA site (z = 3.39, 6 vox at [−46 −56 0]).

**Fig 5:**
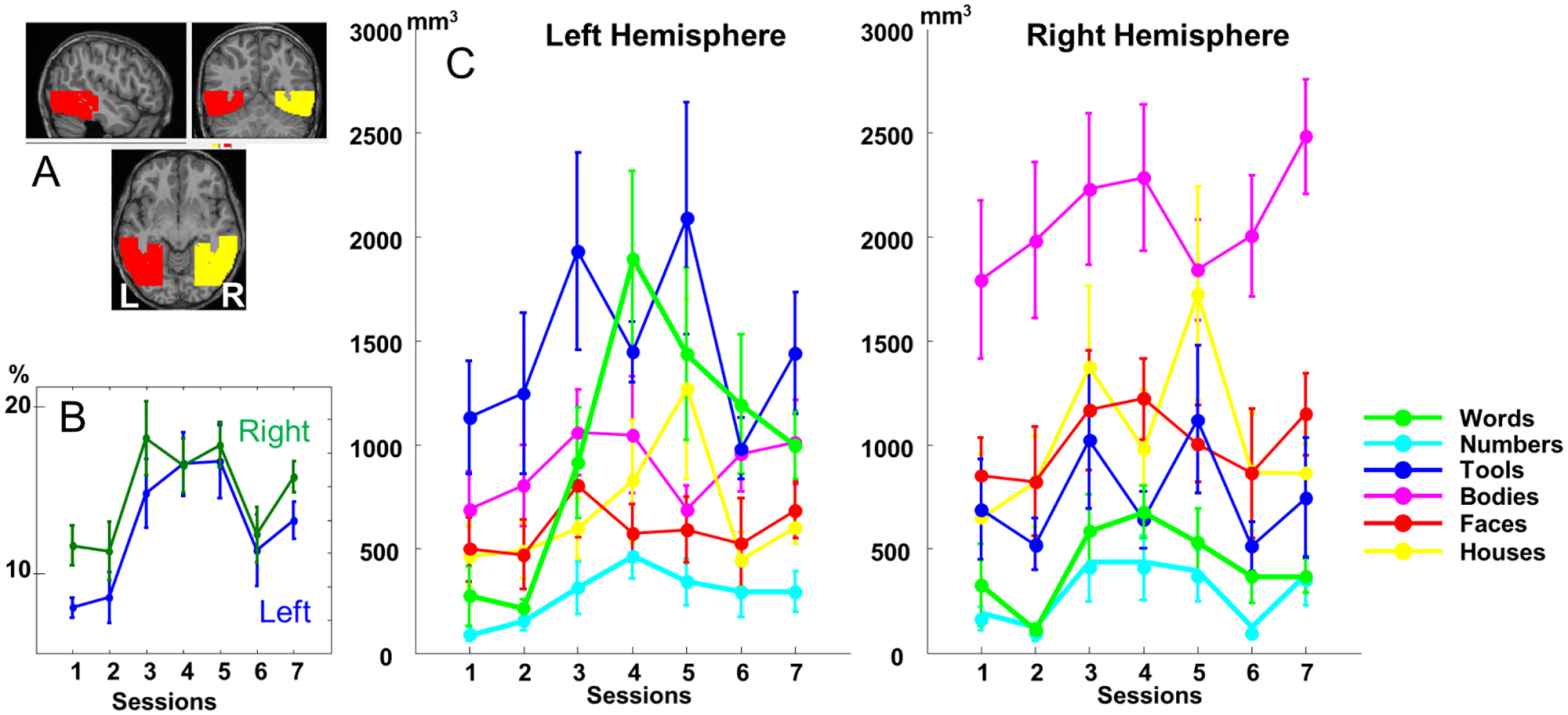
Evolution of the volume of activity evoked by the different categories in ventral visual cortex. (A) Slices showing the analyzed ventral visual region (“ventral mask”). (B) Percentage of voxels showing activity specific to at least 1 visual category (i.e., voxels whose activity was significant at *p <* .001 in the contrast of one category relative to all others). The active volume was expressed, in each child, as a proportion of ventral visual volume (i.e., the intersection of each child’s gray matter mask and the left and right ventral masks). (C) total volume (in mm^3^) of voxels showing significantly more activity for a given category relative to all others (voxelwise *p <* 0.001, uncorrected). For reference, the search regions were 36,080 mm^3^ (left) and 35,896 mm^3^ (right), and the volume expected by chance would therefore be approximately 36 mm^3^. S2 Data.

To further examine individual responses to the different visual categories and how they were modified by reading acquisition, we restricted the next analyses to subject-specific masks encompassing the fusiform region in the left and right hemisphere (Fig 5). First, we asked whether there was a systematic expansion in the number of category-specific voxels, both for reading and for other categories, during the course of reading acquisition. We therefore computed, for each subject and each session, the number of voxels significantly more activated by a given category than by the others (*p <* 0.001; hereafter called “specific voxels” for short). We entered these data into an ANOVA with factors of category (6 levels), sessions (1–6), and hemisphere (left and right). The results revealed a main effect of sessions (F[5, 45] = 5.6, *p <* .001), category (F[5, 45] = 9.0, *p <* .001), hemisphere (F[1, 9] = 10.1, *p =* .011), and an interaction session × category × hemisphere (F[25, 225] = 2.1, *p =* .002), which reflected a significant session × category interaction in the left but not the right fusiform region (Left: F[25, 225] = 1.7, *p =* .024; Right: F[25, 225] <1). Not surprisingly, only words showed a significant change in the number of selective voxels across sessions in the left hemisphere (F[5, 45] = 5.2, *p <* .001), reflecting a sudden increase after session 2, i.e., the start of school. No other visual categories showed a significant change of volume over the 6 sessions (Fig 5), in either hemisphere. The only other significant effects concerned hemispheric differences: there was a left-hemispheric asymmetry for words (F[1, 9] = 16.6, *p =* .003) and tools (F[1, 9] = 32, *p <* .001), whereas bodies (F[1, 9] = 48, *p <* .001), houses (F[1, 9] = 14, *p =* .005), and faces (F[1, 9] = 12.6, *p =* .006) induced larger volumes of activation in the right hemisphere. No effect of hemisphere was seen for numbers (F[1, 9] < 1).

We then asked whether the emergence of the VWFA modified the location of the responses to other categories. We therefore computed, for each image category and for each session, the distance (in mm) of its activation barycenter from the subject’s mean VWFA barycenter determined in sessions 6 and 7 (see Methods) and entered this distance into an ANOVA with category (4 levels, excluding words and numbers) and sessions (1–6). Only the main effect of category was significant (F[3, 27] = 6.61, *p =* .002), reflecting the obvious differences in locations of the activations for each visual category. There was no linear effect of session (F[1,9] < 1), no session × category interaction (F[3, 27] < 1), and no effect of session within each category indicating an absence of change in the position of activations relative to the VWFA. This was also the case when we considered the peaks of activation of houses, bodies, and tools relative to the left and right FFA barycenter, suggesting that the peaks of activation of the visual categories were not displaced by the development of the VWFA. Therefore, the development of the activation to words in the left fusiform region during the first year of school did not affect the volume or location of the activation to other categories.

### Preferences of RR voxels prior to reading acquisition

**Fig 6:**
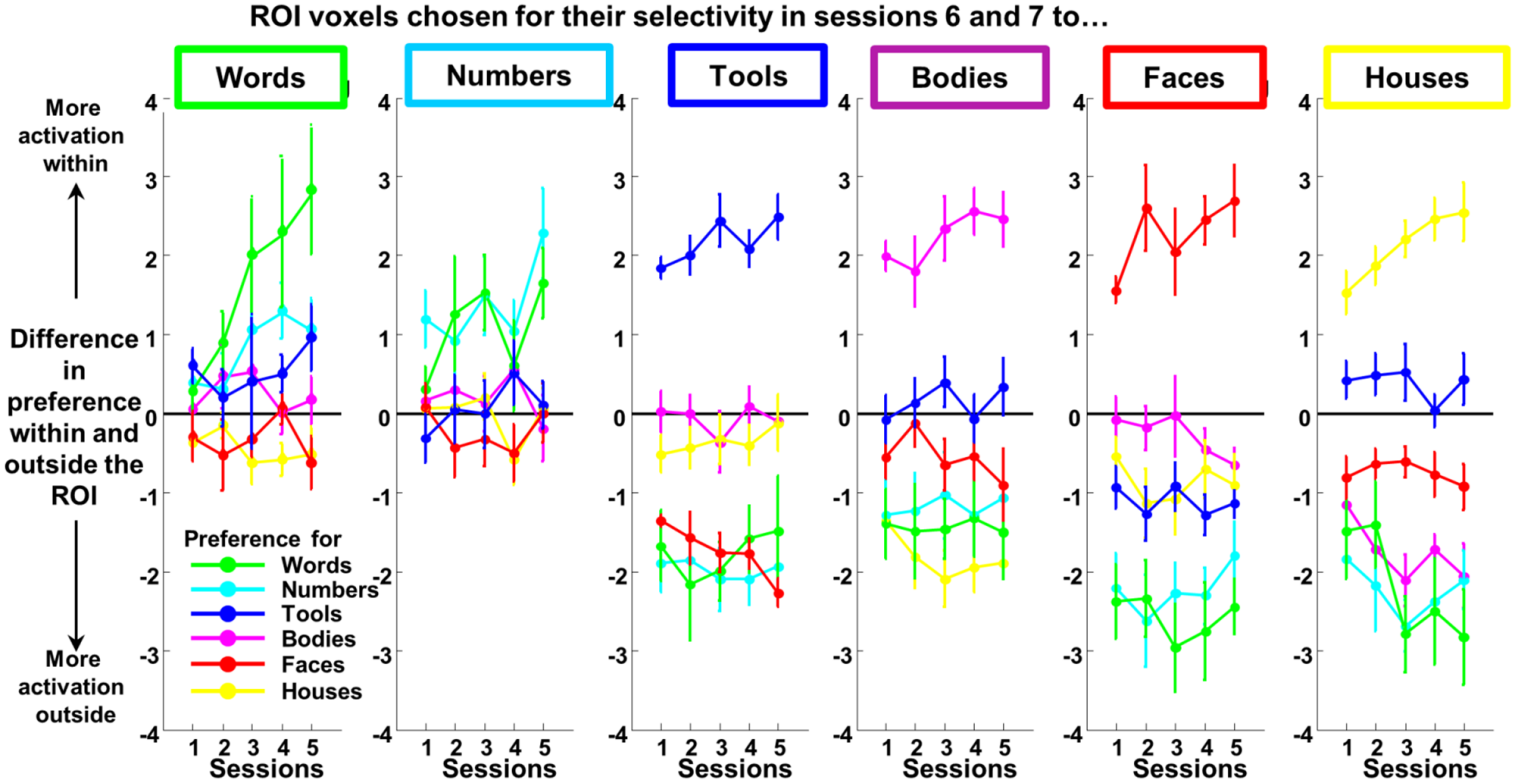
Evolution of responses in left-hemispheric voxels that ultimately specialize for a given category. For each child, we subdivided left ventral temporal voxels into the ROI formed by those selective for a given category during the final sessions 6 and 7 (category > others, *p <* 0.001) and the remaining voxels. We then went back in time and asked how these 2 sets of voxels differed in their responsivity to the various categories of stimuli in the preceding sessions 1–5. Each plot shows the difference in activation evoked by a given stimulus (e.g., words) relative to other categories (excluding words), in voxels within and outside the specified ROI. For instance, the green curve at left indicates that word-selective voxels in sessions 6 and 7 showed no specificity for words during sessions 1 and 2 (values not different from 0) but became selective in sessions 3, 4, and 5 (positive values). Therefore, there is a clear emergence of selectivity for words in RR voxels. The fact that other (nongreen) curves in this panel are indistinguishable from 0 indicates that the VWFA voxels cannot be anticipated by their responsivity to other categories. By contrast, voxels selective to tools, houses, faces, or bodies in sessions 6 and 7 already exhibit their category specificity in the very first sessions. S3 Data. ROI, region of interest; RR, reading-related; VWFA, visual word form area.

We next asked whether the voxels that become specific for written words in sessions 6 and 7 could already be distinguished by a particular response profile in previous sessions (1 to 5) and notably before reading acquisition. Within our ventral mask, we therefore distinguished RR and nRR voxels, using a voxelwise threshold of *p <* 0.001 on the words > other visual categories in sessions 6 and 7. On average, we recovered 172 RR voxels (range 39–388 voxels), i.e., 1,376 mm^3^ representing an average 3.8% of the search volume (range 0.87%–8.57%). We could then go back in time and examine the properties that distinguished RR voxels from nRR voxels in the preceding sessions (Fig 6, left panel).

First, were these voxels already specific for words before school, or did a selectivity for words emerge only after the onset of reading acquisition? A comparison of the mean activation evoked by words versus other categories—in RR versus nRR voxels—revealed no significant difference in session 1, approximately 2 to 3 months before the start of school (t[9] < 1). There was a marginal effect in session 2, which occurred around school onset (t[9] = 2.23, *p =* .053). A clear difference emerged in session 3 (t[9] = 2.73, *p =* .023), session 4 (t[9] = 2.40, *p =* .040), and session 5 (t[9] = 3.46, *p =* .007). Similar findings were found when analyzing only the RR voxels for a difference in responsivity to words versus other categories (S7 Fig, left panel). Those findings show that, even with a sensitive analysis targeted precisely at subject-specific voxels that ultimately become RR, we could not identify an early responsivity to words prior to schooling.

In this respect, words behaved differently from other categories: when the same analysis was replicated with voxels specific for other categories of visual images in sessions 6 and 7 (294 voxels sensitive to tools, 167 to houses, 150 to faces, and 212 to bodies in the left hemisphere), these voxels were already specific in sessions 1 to 5 (Fig 6 and S6 Fig, all *p <* .003). Even for numbers—although a reduced set of voxels showed a preference for numbers than for other categories in sessions 6 and 7 (mean = 22 voxels, range = 1–63)—a difference in activation to numbers versus other categories was already found in all previous sessions except session 2 (*p =* 0.13, for all other sessions, p between 0.025 and <.001). Therefore, only for reading did we see the emergence of a novel cortical selectivity during the first year of schooling (which, in France, focuses almost entirely on reading acquisition).

Second, we asked whether the ultimate preference of RR voxels for words could be anticipated by an early preference of those voxels for another category—thus testing, at the single-subject level, previous suggestions that reading might specifically encroach, for instance, on face-related circuits [14, 15,51]. To this aim, we computed the average activity evoked by each of the 4 nonsymbolic image categories (tools, houses, faces, and bodies) relative to rest in the RR voxels and in each of the 5 initial sessions. We submitted these data to an ANOVA with categories and sessions as within-subject factors. A main effect of category was present (F[3, 27] = 6.91, *p =* .001), indicating that RR voxels responded to other categories in the following order: tools (mean activation = 0.51), bodies (0.32), houses (0.11), and faces (0.09). Posthoc paired *t* tests analyzing each possible pair using Holm correction for multiple comparisons revealed that all pairwise comparisons were significant (ps ≤ .01) except faces versus houses, which had a similar weak level of activation in RR voxels (p > .1), and bodies and tools (*p =* .097), which both produced a larger response in these voxels. Those results indicate that RR voxels showed an initial response to tools and bodies. Importantly, there was no interaction with sessions (F[3, 27] < 1), again indicating that those biases did not change during reading acquisition.

Another important question, however, is whether such preferences are sufficiently unique to RR voxels that they would suffice to determine which voxels ultimately become specialized for reading. We operationalized this question by asking whether RR voxels differed from nRR voxels in their profile of responsivity to nonletter categories (Fig 6). Interestingly, RR voxels, relative to nRR, preferred numbers over other categories early on (Fig 6, left panel, light blue curve). This difference was not significant in sessions 1 (t = 2.25) and 2 (t < 1) but became clear from sessions 3 to 7 (t[9] = 3.10, 3.67, 2.70, 2.6, and 4.62, respectively), suggesting that the development of letter responsivity was accompanied by an additional responsivity to numbers. With respect to other image categories, however, the responses of RR voxels did not differ from those of nRR voxels. Only a small preference for tools tended to be present in RR voxels more than in nRR voxels in session 1 and 5 (t[9] = 2.72, *p =* .024 and t[9] = 2.27, *p =* .049). Therefore, on average, RR voxels were not especially distinguished from nRR voxels in their initial commitment to a specific category, with the possible exception of a small bias for tools.

A related question is whether RR voxels are simply less specialized overall. According to this hypothesis, reading would “land” in voxels that are not already strongly committed to a particular visual category and are therefore available for learning. To evaluate this possibility, for each voxel, we also calculated the F-test quantifying any difference between the 4 nonsymbolic image categories (tools, houses, faces, and bodies). We then examined whether this F-test differed for RR versus nRR voxels. Again, no significant difference was found in any session (ps > .05), indicating that RR voxels were not particularly distinguished by a reduced initial commitment. We did find, however, a small but significant interaction with a linear contrast over sessions 1 through 7 (F[1, 9] = 7.26, *p =* .025), indicating another type of difference between RR and nRR voxels: nRR voxels exhibited a significant linear increase of the F-test, indicating that their selectivity for nonsymbolic image categories increased (F[1, 9] = 15.1, *p =* .004), while this was not true for RR voxels (F[1, 9] = 2.5, *p =* .15).

Those findings led us to ask, symmetrically, what were the initial preferences and temporal evolution of voxels that ultimately preferred nRR categories. When we selected voxels that ultimately preferred tools, faces, houses, bodies, or numbers over the other categories, we found that their preferences were temporally stable (unlike what was found for words): as shown in Fig 6, when going back in time, these voxels already exhibited a strong and significant selectivity for their preferred category in the first scanning session and a corresponding lack of responsivity to their nonpreferred categories. For instance, house-responsive voxels in sessions 6 and 7 showed a strong preference for houses in sessions 1 and 2 (ps < .001), accompanied by a mild response to tools (ps > .05) and a significantly smaller response to faces, bodies, words, and numbers (ps < .035) compared to the responses found in non–house-responsive voxels. These preferences were therefore entrenched early on and remained stable or slightly increased over time (Fig 6), as confirmed by the above F-test. Most importantly, voxels selective for tools, houses, faces, or bodies systematically showed an early negative responsivity to words relative to other categories (Fig 6, green curve, ps < .05). This finding implies that, if a voxel was strongly selective for a category other than words, it tended to be less responsive to written words than other voxels—and this antipreference remained stable over the time course of early reading acquisition.

The above conclusions remained when we performed a number of variants of the above analysis. Namely, the stability of visual preferences in nRR voxels was observed when we selected category-selective voxels in sessions 1 and 2 and evaluated their responses in sessions 3 through 7 (S7 Fig), when we considered the entire set of voxels within anatomically defined regions: fusiform gyrus (FG)1 and FG2 [52], and when we performed the same analyses in the right hemisphere.

### Multivariate patterns of category selectivity

Averaging over an entire set of RR voxels, as we did in the above analyses, may mask the presence of fine-grained activity patterns that are specific to a given subject or a given category [53,54]. In a final analysis, we therefore used multivariate pattern analyses in order to quantify the stability and the evolution of subject-specific activation patterns within the entire ventral mask and, more specifically, within the VWFA.

**Fig 7:**
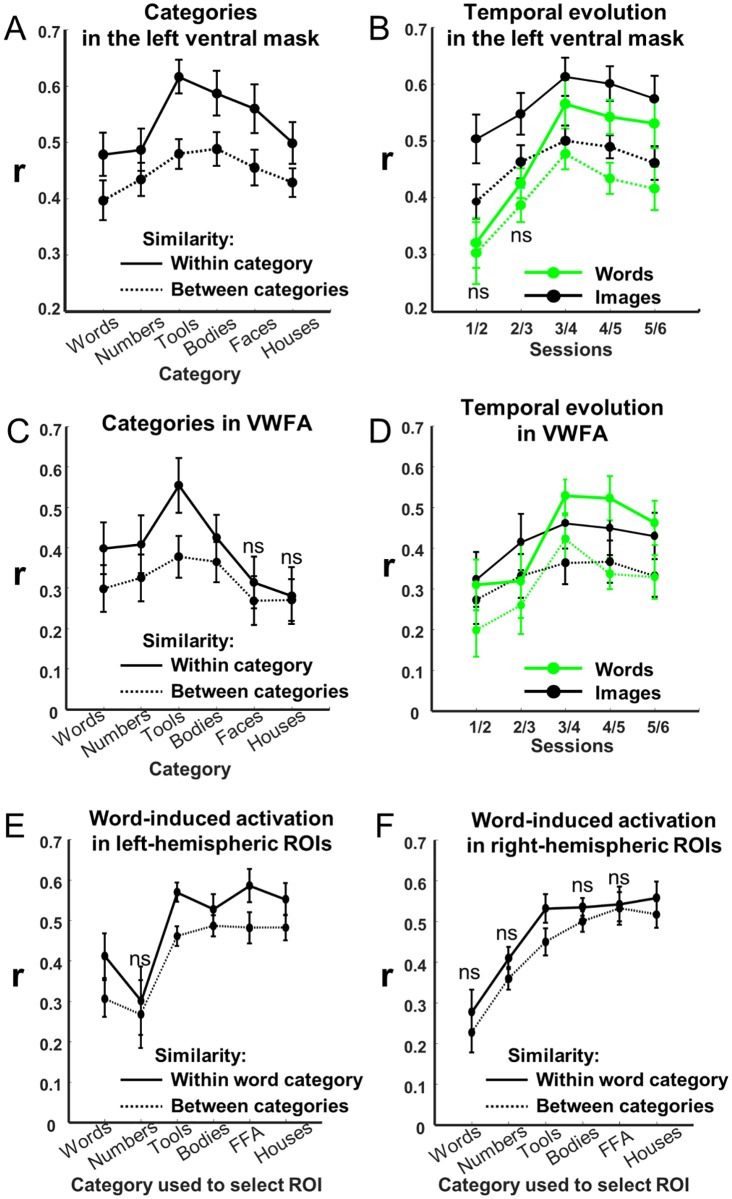
Representation similarity analysis. In each graph, the thick line shows the within-category correlation coefficient r between 2 independently measured activation patterns, measuring the reproducibly of activation patterns in a given area, and the dotted line shows the between-category baseline (r was measured within each subject, then averaged; bar = ±1 standard error). A significant difference between those curves indicates a reliable category-specific activation. (A) When averaging over sessions, reliable patterns were present for all categories in the left ventral mask. (B) Temporal evolution across sessions of the r coefficient for words and for the average of nonsymbolic images (tools, bodies, faces, and houses) in the left ventral mask. (C) and (D), same as (A) and (B), restricted to the visual word form area. (E) and (F), similarity of the word-induced activation pattern in each ROI selective for a given category relative to others, respectively within the left and right ventral masks. All comparisons within versus between category are significant, except those indicated as ns. Underlying data found in S4 Data. FFA, fusiform face area; ns, nonsignificant; ROI, region of interest; VWFA, visual word form area.

We first performed this analysis over the entire ventral mask and probed the reproducibility of the pattern of activation between one session and the next (i.e., over a delay of approximately 2 months) for each category relative to rest (S8 Fig). We performed an ANOVA on the correlation coefficients within category (e.g., correlation between the vectors of activity for faces in 2 successive sessions) versus across categories (e.g., correlation between the vector for faces at session *n* and for houses at session *n* + 1) over the 6 sessions of the first school year. As shown in Fig 7A, the within-category correlation was systematically positive (ranging around approximately 0.5–0.6) and systematically higher than the between-category correlation, indicating a robust replicability of the category-specific activation patterns across successive scanning sessions (left hemisphere: F[1, 9] = 58; right: F[1, 9] = 49, ps < .001). This result was observed for each category in both hemispheres (ps < .007), with the sole exception of words in the right hemisphere (F[1.9] = 3.35, *p =* .1). Interestingly, the activation pattern for bodies (*p =* .004) and faces (*p =* .02) was significantly more reproducible in the right hemisphere than in the left, whereas the reverse was true for words and tools (ps = .008). Therefore, this multivariate analysis revealed that activity patterns could be reliably measured in our young children for all categories.

We next examined how those patterns evolved over sessions (Fig 7B). For nonsymbolic images, this reliability was stable over time, whereas for words (and numbers), the reliability increased between sessions 3 and 4. This conclusion was confirmed by an interaction between category, correlation type, and a linear contrast for sessions (F[5, 45] = 2.6, *p =* .035) in the left ventral visual cortex, due to an interaction between sessions × correlation type for words (F[1, 9] = 6.9, *p =* .028) and numbers (F[1, 9] = 6.99, *p =* .027) but not for the other categories, bodies, houses, faces, and tools (all F[1, 9] < 1).

Armed with this analysis, we could next ask whether the VWFA itself is a site that is initially devoid of category-specific patterns or, on the contrary, whether it is already traversed by reproducible activations for visual categories other than words; and if the latter is true, whether those activations change over time. We therefore performed the same analyses as above on voxels restricted to the VWFA (RR voxels defined on sessions 6 and 7). Unsurprisingly, a reproducible pattern of activation was found for words (F[1, 9] = 15.1, *p =* .004) and numbers (F[1, 9] = 11.4, *p =* .008) but also, remarkably, for nonsymbolic images (F[3, 27] = 27.7, *p <* .001) mainly due to tools (F[1, 9] = 31, *p <* .001) and to a lesser degree bodies (F[1, 9] = 10.8, *p =* .009; no significant effect for houses and faces, ps > .1). Importantly, the pattern reliability for tools was present in sessions 1 and 2 (F[1, 9] = 12, *p =* .007) prior to reading and remained stable over time (interaction with sessions, F < 1). Therefore, RR-related voxels contain reliable multivariate activity patterns for visual categories other than words, and the emergence of reliable activation to words (Fig 7D) in the course of reading acquisition did not occur at the expense of other categories.

Similar results were observed for the other category-specific ROIs (defined on sessions 6 and 7). Most importantly, the pattern of activation for words was reproducible in the tool ROIs (left and right, ps < .001), house ROIs (left: *p =* .011; right: *p =* .005), left FFA (*p <* .001), and left-body ROI (*p =* .02) (Fig 7E and 7F). Therefore, activation patterns were distributed beyond the strict boundaries of their significant category-selective clusters [53]. As discussed below, those results are compatible with a superposition principle according to which visual categories are encoded by overlapping activity patterns over the same voxels.

## Discussion

### Summary of the main results

In 10 individual children, we visualized the emergence of reading circuits by reproducibly scanning them with a battery of visual stimuli during, and 1 year after, the first year of schooling. Prior to schooling, the VWFA could not be detected, although selectivity for faces, houses, bodies, or tools was clearly present. Within the first 2 to 4 months of schooling, the VWFA emerged at the group level and in 8 out of 10 individual children (in the remaining 2, activation was present and significant at the voxel level but not after correction for multiple comparisons at the whole-brain level). The VWFA immediately appeared at its adult location, with a subject-specific topography that was stable over time. An inverted-U curve indicated that—in most of the reading circuit, including anterior VWFA and posterior parietal cortex—the onset of schooling was associated with a peak of activation that then slightly receded over the following months.

Most importantly for theories of reading acquisition, the data allowed us to retrospectively examine what the VWFA voxels were responding to prior to reading acquisition. Reading did not recruit voxels that were selective for faces but systematically encroached on slightly more lateral sectors of cortex, within the left occipitotemporal sulcus, in a region that was not strongly selective to any category but did respond more to tools than to other visual categories. The emergence of the VWFA occurred without radically altering the preferences or the topographical organization of ventral visual responses to faces, bodies, houses, or tools, although at the group level we detected a positive correlation between reading performance and the amount of right fusiform activation to faces.

Those results suggest that the VWFA emerges quickly, at a fixed and constrained location within a well-organized mosaic of preferences, and by superimposing itself onto existing preferences while minimally altering them. These points, which constrain theories of brain development, will now be discussed in turn.

### Fast emergence of the VWFA

The first question that our study aimed to answer is how quickly the VWFA emerges in children during the course of reading acquisition. In our subjects, the VWFA was undetectable prior to schooling: in our first 2 scans, we observed no specific response to letter strings relative to other visual categories. This is a negative result and should therefore be taken cautiously, but it is strengthened by the fact that we could easily detect reliable preferences for all nonsymbolic visual categories (faces, houses, bodies, and tools). The absence of the VWFA could be due to the fact that we selected children with very little prior knowledge of letters (Fig 1) in order to control their exposition to letters and reading when they entered school. Other studies show that preschoolers who possess some knowledge of letters already exhibit letter-specific steady-state responses that are detectable in a few minutes of electroencephalography (EEG) recording [22].

Our results suggest that, by 2 to 4 months after school onset, a change in the volume of selective activation to words is already detectable (Fig 5) and is associated with the establishment of a stable pattern of subject-specific activation for words relative to other categories (Fig 7). This finding is entirely coherent with another longitudinal study [17] in which left occipitotemporal responses emerged in fMRI and event-related potentials once preschoolers had been exposed to a few weeks of training with the GraphoGame, a software game that teaches grapheme−phoneme correspondences. Lochy et al. (2016) and Maurer et al. [21,55,56] likewise observed a rapid growth of left-lateralized event-related potentials evoked by letter strings (N170) in the course of reading acquisition, both in children and in adults acquiring a new script. Our results are compatible with those prior findings in as much as they show that VWFA responses emerge within the first few months of reading acquisition.

Although reading acquisition was necessarily confounded with age in the present within-subject study, we still observed an effect of reading speed on the VWFA activation even when the effect of age was taken into account (Fig 4A). In other between-subject studies, the 2 variables have been clearly decorrelated, and the results indicate that age alone does not suffice to induce the observed changes. For an equal age of approximately 6 years, children who have already learned to read by themselves prior to formal schooling already exhibit a specialized VWFA, contrary to those who have not [57]. Even adults, in the absence of schooling, do not develop a specific ventral visual response to letter strings relative to other visual categories [14,23].

The remarkable speed with which word-specific responses emerged in 6-year-old children fits with other findings indicating a high degree of plasticity in children’s ventral visual cortex. Indeed, the fusiform cytoarchitectonic area FG2, where the FFA and VWFA are located, shows a prolonged development and does not fully mature until late childhood, in contrast with the nearby area FG1 [33]. Its plasticity supports not only reading acquisition but also other forms of visual expertise. For instance, the acquisition of music reading, starting at 3 to 4 years of age, has been shown to induce a large activation of lateral ventral temporal cortex to printed music in professional musicians and to induce a displacement of the nearby VWFA [6]. In adults, fusiform plasticity is sufficient to acquire a novel expertise for birds and cars [58], greebles [59,60], children’s faces in teachers [61], or Braille reading in nonblind adults [62]. Its plasticity might be reduced in adults compared to 6-year-olds. In exilliterate subjects who learned to read as adults, the VWFA is present but only moderately activated in proportion to reading speed, and written words induce a coarser pattern of activation extending into bilateral ventral visual regions [14]. In a recent single-case study [63], we used longitudinal fMRI in a single illiterate subject to follow the trajectory of adult literacy acquisition over the course of 2 years. Unlike the steep nonlinear increase shown here in children, this person’s VWFA emerged slowly and with a continuous increase over a period of several months, paralleling a slow increase in behavioral reading ability. Therefore, the fast changes induced by reading acquisition that we observed here betray the intense plasticity of children’s immature ventral occipitotemporal cortex. This conclusion is also confirmed by a recent animal model of symbol recognition that shows that, while both juvenile and adult monkeys can acquire a behavioral ability to recognize Arabic numerals and letters, only the juveniles develop a category-specific response to such symbols in inferotemporal cortex [64].

### Developmental trajectory of the reading circuit

Most previous studies of reading acquisition have either been cross-sectional [14,23,65–67] or longitudinal with few data points spread over a long time period, typically 6 months to several years [19,21,68,69]. Here, we aimed to clarify the developmental trajectory of the VWFA and other areas of the reading circuit by obtaining detailed single-subject measurements spaced every 2 months around the onset of literacy. Theories of child development have contrasted several accounts of the emergence of functional brain specialization (reviewed in [70]): according to a maturational account, the adult reading circuit should progressively emerge at its normal location, with increasing size and intensity as increasingly larger populations of neurons become tuned to written words. An alternative account, the interactive specialization model, predicts that the reading circuit should be initially disorganized, diffuse, distributed over broad cortical territories, and that it should dynamically change in a stochastic manner until it settles into a minimal set of regions the interactions of which optimally fit the task. Finally, the skill-learning model postulates an initial phase of effortful processing, relying on nonspecific prefrontal and parietal areas, followed by a progressive reduction of this activity and its transfer to specialized posterior areas.

Our data show features of both the maturational and the skill-learning models. On the one hand, the VWFA landed immediately at its adult-like location: just 2 months after school onset, it was already detectable, at its final location (e.g., Fig 3), and with a fine-grained topographic pattern that showed little or no change over the next 1.5 years (Fig 7D). Those findings argue against a stochastic search or dynamic change in interregional interactions and instead suggest the rapid maturation of a highly constrained brain circuit (the nature of those constraints is discussed below). On the other hand, several regions of the reading circuit showed an inverted-U activation as a function of time, peaking around the first year of schooling (Figs 4 and 5) and then decreasing steadily. In fact, activation at several parietal and inferior frontal sites eventually decreased down to an undetectable level (Fig 3). In the left occipitotemporal cortex, the volume of RR activation increased sharply and then was nearly halved (Fig 5C). This volume change arose because activation in the posterior part of the VWFA followed an inverted-U pattern (Fig 2), while activation at the peak of the VWFA proper steadily increased in parallel to behavior (Figs 2 and 6). In other words, there was a progressive concentration of activation in a specific ventral area and a progressive disengagement of parietoprefrontal areas, in agreement with the skill-learning model.

This developmental pattern fits with previous evidence that reading progressively switches from a slow, serial, effortful mode to a fast, parallel, efficient mode. The posterior parietal cortex, in particular, participates in a top-down attention circuit that has been implicated in letter-by-letter reading, both in adult patients with pure alexia following lesion at or near the VWFA [71] and in normal adult readers confronted with unusual formats such as vertical or s t r e t c h e d words [72]. Its strong activation early on in literacy acquisition is therefore congruent with the classic observation that young readers show a strong effect of word length on reading time [73,74]. This effect progressively vanishes as reading becomes automatized and may therefore explain the inverted-U profile of parietal activity that we observed.

The present data thus confirm the existence of a parietal circuit for effortful reading and underline its transient but important contribution during the early phase of reading acquisition. It fits with the suggestion that some children with developmental reading deficits suffer from an “attentional” subtype of dyslexia that prevents them from focusing on each letter in a sequential left-to-right order and avoiding illusions, conjunctions, and letter migrations from previous words [75–78]. In the future, it would be interesting to use the present fMRI task with subtypes of dyslexic children and examine whether the attentional subtype shows disorganized parietal activation while the more classical phonological subtype shows left temporal impairments, as shown in Peyrin et al. [79].

### Factors that determine the location of the VWFA

Another goal of our study was to examine whether a specific profile of functional brain activity predicts which voxels are going to become specialized for written words during reading acquisition. By identifying the VWFA approximately 1.5 years after the onset of schooling and then examining its activity in prior fMRI sessions, we could ask this question within individual subjects at a single-voxel resolution. This is an important improvement over our previous between-subject study of literacy [14], in which intersubject averaging could have caused an artificial overlap between unrelated areas.

The results indicated that, prior to schooling, the voxels that will ultimately become the VWFA are not very strongly specialized for faces, bodies, houses, or tools (Fig 6 and S6 Fig). They are clearly unresponsive to faces and show only a modest response to tools, clearly smaller than the peak response found at a slightly posterior site. Therefore, the VWFA lands at an initially relatively uncommitted cortical site, the main functional characteristic of which seems to be a low responsivity to pictures. This finding suggests that ventral visual cortex becomes progressively committed, first to visual categories such as faces and places [30] and later to cultural acquisitions such as letters and numbers and that the latter wave of cortical specialization is constrained to sites that were left partially or totally uncommitted by the first wave.

Additionally, the present findings confirm that the VWFA emerges at a systematic location relative to other functional landmarks, i.e., lateral to the fusiform face responses (as previously reported in [80]) and overlapping with the most anterior part of the lateral object-responsive cortex (Fig 2). Because the VWFA location is so reproducible, factors other than the mere lack of commitment to other categories are likely to play an important role in its spatial delineation [38]. Candidate factors that have been identified in other studies include (1) cytoarchitectony and extended plasticity [33], (2) preferred connectivity to a dedicated set of left-hemispheric targets that include classical spoken language areas [18,36,81], (3) high-resolution foveal bias [26], (4) preference for high spatial frequencies [82], (5) preference for line junctions that are characteristic of letter shapes [37], and (6) efficient detection of invariant shape features [38,83]. Animal studies in which juvenile monkeys were trained to recognize letters, cartoon faces, or tetris-like pentominos indicate that each of these categories landed at a dedicated site that was reproducible across monkeys, suggesting the existence of a biased proto-organization prior to learning [84]. Human adults are able to learn a new script based on human faces and activate the left, not the right, fusiform region for the facefont words, confirming the strong left bias for phonetic-based script, but they were less efficient than the group trained with Korean fonts, and the location of the activation was slightly displaced in an anteromedial position relative to the location of the VWFA [85].

### Effect of literacy on the ventral visual mosaic

A third goal of our study was to probe the organization of ventral visual cortex prior to reading and to examine how it is changed by literacy. As a selectivity for words emerges in occipitotemporal cortex, does it lead to a reorganization of the responses to other categories?

By 5 to 6 years of age, our results indicate that the mosaic of specialization for visual categories that has been reported in adults [4,86] is already largely in place and changes very little in the course of almost 2 years. There was an initial controversy as to whether the cortical representation of faces—as opposed to that of objects and houses/landscapes—develops relatively late in childhood [27,28] or, on the contrary, is present early on [29,30]. Our results concur with the latter studies in showing that, by the age of 5, category selectivity is already deeply entrenched. In fact, a recent study showed a clear difference in responses to faces versus places in 3- to 8-month-old infants [30], and this was corroborated by a longitudinal fMRI study of juvenile monkeys [87]. In both studies, however, the selectivity was initially imperfect, for instance, showing no clear selectivity for faces over objects in human infants, and the longitudinal study in monkeys indicated that highly selective face patches emerged during the first year of life [87].

Once these regions are in place, do they change with reading acquisition? Our results uncover that, in fact, they are remarkably stable over time. Word selectivity appears against a background of largely unchanged responsivity in nearby voxels specialized for faces, bodies, tools, or places (Figs 2 and 6). Even within the same voxels that ultimately become the VWFA, multivariate responses to tools stay essentially unchanged during literacy acquisition (Fig 7).

The results therefore strongly question the hypothesis that words and faces share the very same cortical circuitry [51,88]. There is, in fact, much evidence that face and word recognition engage nearby but systematically distinct cortical sectors in normal adult readers [14,80]. Neuropsychological dissociations have also been reported between both domains, both in adult subjects [89–92] and in developmental cases [93,94]. The present study is, we believe, the first to directly demonstrate that orthographic representations develop in ventral visual cortex without encroaching directly on preexisting face cortex but rather by invading nearby but more lateral and relatively uncommitted ventral visual cortex.

The results are, however, compatible with a broader view of cortical competition and neuronal recycling [35]. According to this model, word and face recognition emerge at distinct cortical locations, possibly because of their distinct connections to distant areas [18,36], but they both rely on a similar hierarchical architecture common to all ventral visual cortex and therefore compete for expansion into the same overall region of cortex during development [14,35]. During the acquisition of literacy, the VWFA emerges at a site just lateral to the FFA, thus blocking the further growth of face responses in the left hemisphere and forcing them to preferentially develop in a right-lateralized manner [14,15]. Several experimental results fit with this view. First, Golarai et al. [28] observed that, across development, the peak FFA activation evoked by faces does not change with age in either hemisphere. What increases with age is the activation evoked by faces in peripheral voxels within concentric shells that center on the FFA peak—the FFA slowly expands. Second, in a previous study of adult literacy [14], we found that, in adults with variable degrees of schooling and literacy, greater reading scores were associated with a small decrease of face responses at the usual left-hemispheric site of the VWFA. It is crucial to remember that this finding was based on a group comparison and therefore does not contradict the present within-subject results. Finer-grained analysis, inspired by Golarai et al. [28], in fact revealed that the peak response to faces was unaffected by literacy: it was only in voxels that lay at a distance of 8 or 12 mm from the FFA peak that a difference between literates and illiterates was observed (Figure S6 in [14]), again suggesting that cortical competition occurs at the periphery, where literacy shifts cortical boundaries. Third and finally, our adult study also showed a highly significant increase in right-lateralized fusiform activation to faces as the literacy score increased, suggesting that literacy competed with the development of face responses in the left hemisphere [14]. This finding was also observed in 9-year-old normal readers compared to dyslexics [15] and replicated in the present study (Fig 4). It is corroborated by several behavioral and event-related potential studies (for a review, see [13,23,40,41]).

Our study also probed the development of responses to written Arabic numerals. We expected to observe the emergence of number-related responses lateral to the VWFA, at a site known as the visual number form area (VNFA) and more responsive to digits than to letters in educated adults [7,8,95]. The results, however, were weak. There was no evidence for the emergence of any region specialized for numbers. Instead, we found that ventral visual voxels responsive to numbers were also responsive to letters (Fig 6, S3 and S6 Figs). Reading speed correlated with an increased fMRI response to numbers but only at an occipital site posterior to the VWFA (Fig 4). The fact that we observed no clear separation between number-form and letter-form areas, as it was also observed in younger 4-year-old children in Cantlon et al. [29], nor any intraparietal activation specific to numbers is compatible with the hypothesis that, at a young age, digits are primarily processed as readable letter-like symbols but not as meaningful quantities. At least, such quantity processing does not seem to be automatically engaged when the task does not require any calculation but a mere intruder detection (“find Waldo”) as was the case here. Furthermore, we used 4-digit numbers (matched in length to the words) that probably challenged children’s quantity comprehension. Our results are fully compatible with behavioral studies of the number-size interference effect, which indicates an absence of automatic quantity processing in first graders [96].

Finally, beyond the overlap of numbers and letters, our results on visual categories clearly indicate that, in our children, category selectivity is not absolute. Even within subject-specific voxels that were defined by their selective response to one category relative to all others, there were highly detectable differences between the other nonpreferred categories, both in average activation (Fig 6 and S6 Fig) and in multivariate activation pattern (Fig 7 and S8 Fig). This result, which was first observed in adults [53], suggests that at the relatively modest resolution used here (3 mm isotropic), neural responses to different visual categories are spatially intermixed. It does not, however, exclude that entirely selective patches would emerge in higher-resolution scans, particularly for faces [97].

### A revised cortical recycling model for reading

**Fig 8:**
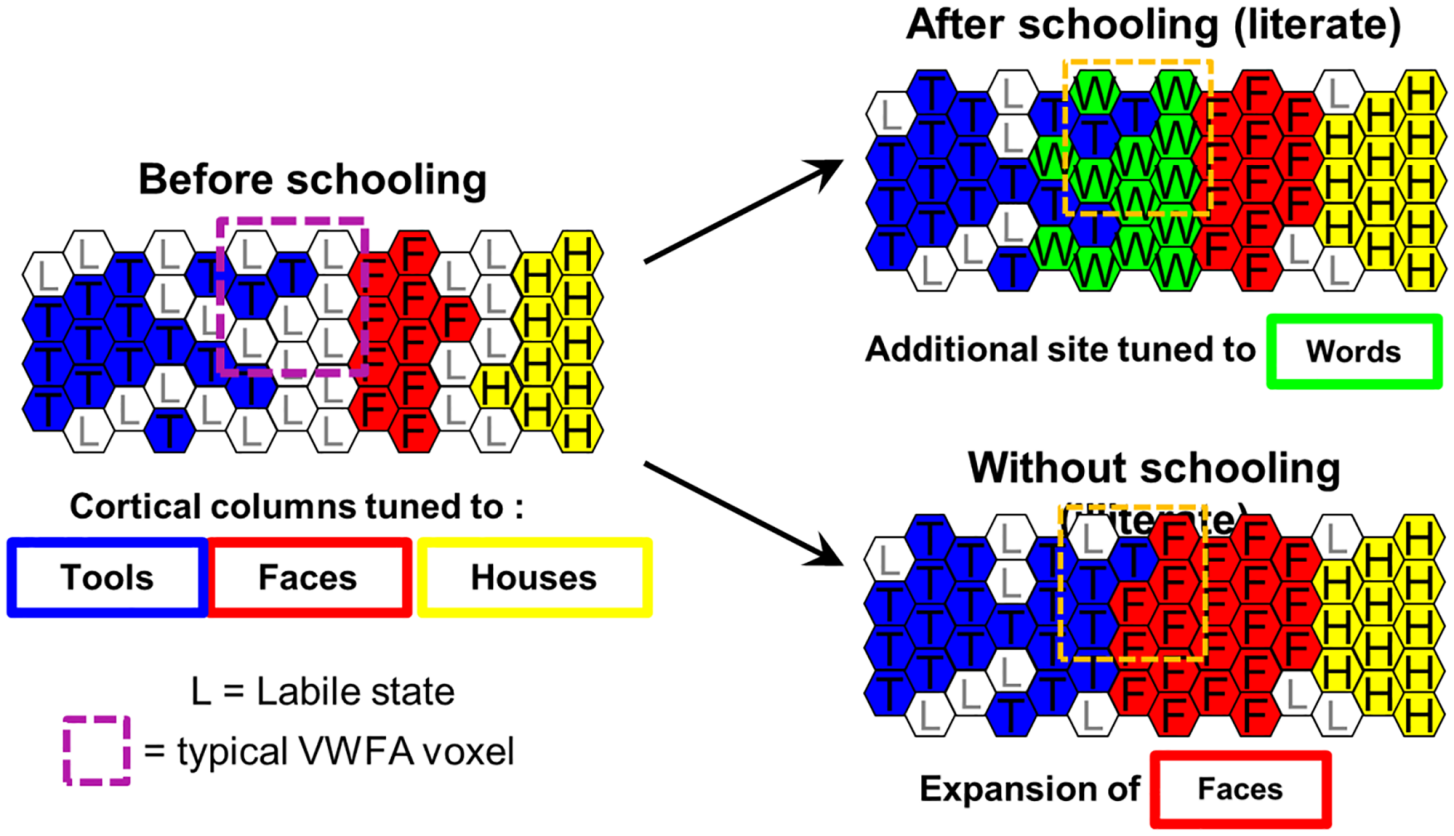
Schematic model of ventral visual development. Hexagons depict cortical patches or columns specialized for a given category (tools, faces, houses) or in an uncommitted labile state. Each group of hexagons schematizes the state of the left-hemispheric ventral visual mosaic at a given age. Prior to schooling (left), some columns are already committed to a given category, with a systematic lateral-to-mesial organization (left-to-right), but many are still labile. When schooling starts, some columns commit to written words (top right). In the absence of schooling, the same columns are progressively invaded by nearby representations of tools and faces (bottom right). The dashed yellow box illustrates how a single fMRI voxel may comprise a mixture of cortical responses to tools and words. The model can explain why, (1) in literate children, VWFA voxels become selective to words while maintaining their prior response to tools; (2) literacy blocks the expansion of face responses in the left hemisphere, restricting their growth to the right hemisphere; and (3) in illiterate subjects, relative to literate ones, face responses are larger in the left hemisphere and thus less asymmetrical in favor of the right hemisphere. F, faces; fMRI, functional magnetic resonance imaging; H, houses; L, labile; T, tools; VWFA, visual word form area.

Fig 8 presents a schematic model of the emergence of ventral occipitotemporal organization in relation to literacy. This model is meant as a graphic summary of our findings and an illustration capable of accounting for the present and several earlier observations within a single framework [14,28–30,53,98–101].

The model starts with the observation that, prior to schooling, category selectivity for faces, houses, and tools is already present (as well as responses to bodies, which are omitted from the Figure for simplicity). Such macroscopic category-selective fMRI responses probably arise from the existence of columns or patches of neurons responsive to each category and/or its visual features and clustered in a reproducible sector of cortex. Indeed, the existence of such dedicated cortical patches was directly demonstrated in monkeys (e.g., [97,99,101]). Developmental fMRI in humans [27–30] and monkeys [87] suggests that such category selectivity emerges within the first year of life and progressively expands in subsequent years. Therefore, we postulate that, prior to reading, the child’s ventral visual cortex comprises a mosaic of specialized sites as well as other more labile sites (respectively appearing in color and in gray in Fig 8).

The key result of the present study is that, with education to literacy, responses to written words emerge at a fixed location within this mosaic (the VWFA), at initially weakly specialized sites, and without altering their (weak) preexisting responsivity to other images. This is captured in Fig 8 by assuming that words invade the patches that were left labile at the time of schooling and lie near those already activated by objects and faces. Within the same fMRI voxel, we may therefore find patches of neurons selective for words and for other images, particularly objects. This “superposition principle” may explain why, throughout the emergence of the VWFA, across 7 scans, we continuously remain able to decode a stable representation of objects within the same voxels (Fig 7B and 7D).

For simplicity of illustration, the superposition principle is illustrated in Fig 8 at the level of adjacent patches, each specializing for a given category. However, superposition may also exist within the same populations of neurons. Neurophysiological studies have revealed neuronal vector codes whereby the same neurons may be engaged in the simultaneous coding of several features of the stimuli along orthogonal vector dimensions [102,103], compatible with the hypothesis that a given neural population may contain multiple superimposed or multiplexed codes in a high-dimensional space [86,104,105]. According to this view, word responsivity may emerge through the progressive differentiation of new principal axes within a preexisting neural population, without altering its pattern of responsivity to other categories. Future studies could use high-resolution fMRI (<1 mm) to attempt to separate the two models of cortical specialization (dedicated patches versus overlapping vectors).

Fig 8 also illustrates why, in the course of the development, superposition may give way to cortical competition. In literate children, the progressive dedication of an increasing number of neuronal patches to written words progressively prevents the expansion of the nearby object and face patches, which occurs in illiterates in the absence of schooling. The model nicely explains how (1) the VWFA emerges at a cortical site that is unresponsive to faces in young children; and yet (2) at a later age, this site shows a greater response to faces in illiterates than in literate subjects [14] and in dyslexics who are not able to acquire fluent reading than in normal 9-year-old readers [15].

### Conclusion

Our data indicate a remarkable degree of stability and reproducible order in the organization of children’s ventral visual cortex in at least 2 respects. First, prior to reading, the ventral mosaic of preferences for faces, houses, tools, and bodies is already in place and reproducible across individuals; and second, during reading acquisition, word responses quickly emerge at a reproducible location distinct from those already committed to other preexisting categories. Such reproducibility implies that the architecture of human ventral visual cortex must be strongly preconstrained, and the precise identification of those constraints, whether cytoarchitectural or connectional, is an important goal for further research. Given such constraints, it should perhaps not be surprising that a novel visual category as different as letter strings, which involves specific computational and connectivity requirements, lands at its own distinct location. We may also reverse the reasoning and wonder whether the shapes of the most successful cultural objects, such as letters and numbers, were selected across cultural evolution in order to be quickly learnable [34]. If so, one constraint might have been to avoid direct cortical overlap with any preestablished categories such as faces or tools (see [85] for a nice example in adults trained to a new script). Therefore, biological constraints may explain why the evolution of scripts systematically involves a progressive abstraction away from iconicity and towards abstract shapes (e.g., from hieroglyphic to demotic in ancient Egypt). The success of education might also rely on the right timing to benefit from the highest neural plasticity. Our results might also explain why numerous academic curricula, even in ancient civilizations [106], propose to teach reading around 7 years.

## Supporting information

S1 Table Word-specific visual activations (i.e., words versus other images except grids) in sessions 6 and 7 (or 6 alone for 2 children) in each of the 10 children ordered from the best to the worst reader as determined by their LUM score at the last session. LUM, “Lecture en une minute.”
(DOCX)

S1 Fig Movement data. Average across participants of the maximal movement between volumes in each session. The error bars represent the standard deviation. (A) Translational movements. (B) Rotational movements. S5 Data.
(TIF)

S2 Fig Sagittal and horizontal glass-brain views of the category-specific activations, pooled over all 7 sessions and 10 children. Each category was contrasted with the other pictures (omitting the grids). No significant cluster was observed for numbers. The red arrow indicates the global maximum for a given analysis. Bottom views (B) show additional contrasts of interest. L, left; R, right.
(TIF)

S3 Fig Category-specific activations in an individual child (child 2). Each category is contrasted relative to the other pictures (omitting the grids), pooling across all 7 sessions in the first row and in each of the 7 sessions in the following rows (*p <* 0.001, FWE-corrected at the cluster level at *p <* .05). The scanning dates are indicated in days relative to the first day of school. The reading network (in green) is evident from session 3 (53 days after the onset of school) with a strong parietal and frontal component all along the first year of school (see also Fig 3). Note the response to numbers in session 5, which superposes to words in the left fusiform and bilateral parietal regions. In this child, activations to faces were clear and stable in the ventral areas, whereas activations to houses, tools, and bodies were less visible in each session—but the mosaic of category-specific regions is clearly organized when the statistical power is increased by pooling all sessions together (top row). FWE, family-wise error.
(TIF)

S4 Fig Individual brain flat maps showing the activation to words versus other categories in sessions 6 and 7 (or session 6 alone for 2 children). Children 1 to 5. The children are ordered from the best to the worst reader as determined by their LUM score at the last session (voxelwise *p <* 0.001 and clusterwise *p <* 0.05, FWE corrected). The red dotted line is placed along the left superior temporal sulcus. FWE, family-wise error; LUM, “Lecture en une minute.”
(TIF)

S5 Fig Individual brain flat maps showing the activation to words versus other categories in sessions 6 and 7 (or session 6 alone for 2 children). Children 6 to 10.
(TIF)

S6 Fig Evolution of responses in left-hemispheric voxels that ultimately specialize for a given category. Same as Fig 6, except that the value being plotted is the contrast of one category minus the other picture stimuli (i.e., houses, faces, bodies, and tools), within the specific ROI determined in sessions 6 and 7 for the visual category indicated on the top of each panel. S3 Data. ROI, region of interest.
(TIF)

S7 Fig Fate of left-hemispheric voxels that are initially specialized for a given category. For each child, we identified the left ventral temporal voxels that were selective for a given category during session 1 and 2 (prior to schooling). At this age, no specialization was observed for words and numbers. The curves show how the responses of these voxels to the preferred and nonpreferred categories evolved in subsequent sessions 3 through 7 (same format as Fig 6). S6 Data.
(TIF)

S8 Fig Average similarity matrices between stimulus categories and successive sessions, in the left and right ventral mask and in the left VWFA. The t value of each contrast for one category versus all others was recovered in each voxel of the mask in each infant and each session. These images were then treated as a vector over voxels, and the correlations of these vectors were computed and averaged across subjects. Several effects can be seen: (1) a square pattern along the main diagonal shows a high similarity within each session; (2) diagonals in the adjacent squares demonstrate that the maps of activations are remarkably stable between one session and the next; (3) reproducible reddish squares illustrate that some categories induce similar activation patterns, e.g., bodies and tools in the left ventral mask or bodies, tools, and faces in the right ventral mask; (4) the activations evoked by words and numbers in sessions 1 and 2 are not correlated with those in the following sessions (2 blue lines at the top of the matrix). Stable patterns emerge after session 3. The activation pattern is roughly similar in the subject-specific VWFA (mask of the contrast words > others in sessions 6 and 7, voxel *p <* .001). B, bodies; F, faces; H, houses; N, numbers; S2–S7, sessions 2 to 7; T, tools; VWFA, visual word form area; W, words.
(TIF)

S1 Data Data for Fig 1. (XLSX)

S2 Data Data for Fig 5. (MAT)

S3 Data Data for Fig 6 and S6 Fig. (MAT)

S4 Data Data for Fig 7. (XLSX)

S5 Data Data for S1 Fig. (XLSX)

S6 Data Data for S7 Fig. (MAT)

## Abbreviations

DTI: diffusion tensor imaging
EEG: electroencephalogram
FDR: false discovery rate
FFA: fusiform face area
FG: fusiform gyrus
fMRI: functional magnetic resonance imaging
FWE: family-wise error
IFGoper: inferior frontal gyrus opercularis
IFGtri: inferior frontal gyrus triangularis
LUM: “Lecture en une minute”
MNI: Montreal Neurological Institute
nRR: non–reading-related
ns: nonsignificant
*P*_FWEcor_: p family-wise error–corrected
pSTS: posterior superior temporal sulcus
RAN: rapid automatic naming of pictures
ROI: region of interest
RR: reading-related
RT: reaction time
VNFA: visual number form area
VWFA: visual word form area
