# Supplementary material for: The emergence of the visual word form: Longitudinal evolution of category-specific ventral visual areas during reading acquisition
Source: PLoS Biol. 2018 Mar 6;16(3):e2004103. doi: 10.1371/journal.pbio.2004103 (PMC5856411; doi:10.1371/journal.pbio.2004103)
Supplement: S1 Table — LUM, “Lecture en une minute.” (DOCX) [file pbio.2004103.s001.docx]

| **Child** | **MNI coordinates** | **Z score** | **Cluster size** | **P_FWEcor_** |
| --- | --- | --- | --- | --- |
|  |  |  |  |  |
|  | **x y z** |  |  |  |
| 1 | -48 -68 - 8 | 7.80 | 119 | <0.001 |
| 2 | -42 -62 - 8 | 8.30 | 227 | <0.001 |
| 3 | -50 -62 -16 | 5.67 | 40 | 0.092 |
| 4 | -50 -62 - 8 | 7.62 | 125 | <0.001 |
| 5 | -56 -60 -10 | 7.80 | 303 | <0.001 |
| 6 | -44 -64 - 4 | 7.50 | 157 | <0.001 |
| 7 | -62 -46 -14 | 4.12 | 42 | 0.039 |
| 8 | -44 -42 -16 | 5.13 | 58 | <0.001 |
| 9 | -16 -94 -10 | 5.88 | 83 | <0.001 |
| 10 | -52 -58 -16 | 6.10 | 104 | <0.001 |
