# Supplementary figures and images for: The emergence of the visual word form: Longitudinal evolution of category-specific ventral visual areas during reading acquisition
Source: PLoS Biol. 2018 Mar 6;16(3):e2004103. doi: 10.1371/journal.pbio.2004103 (PMC5856411; doi:10.1371/journal.pbio.2004103)

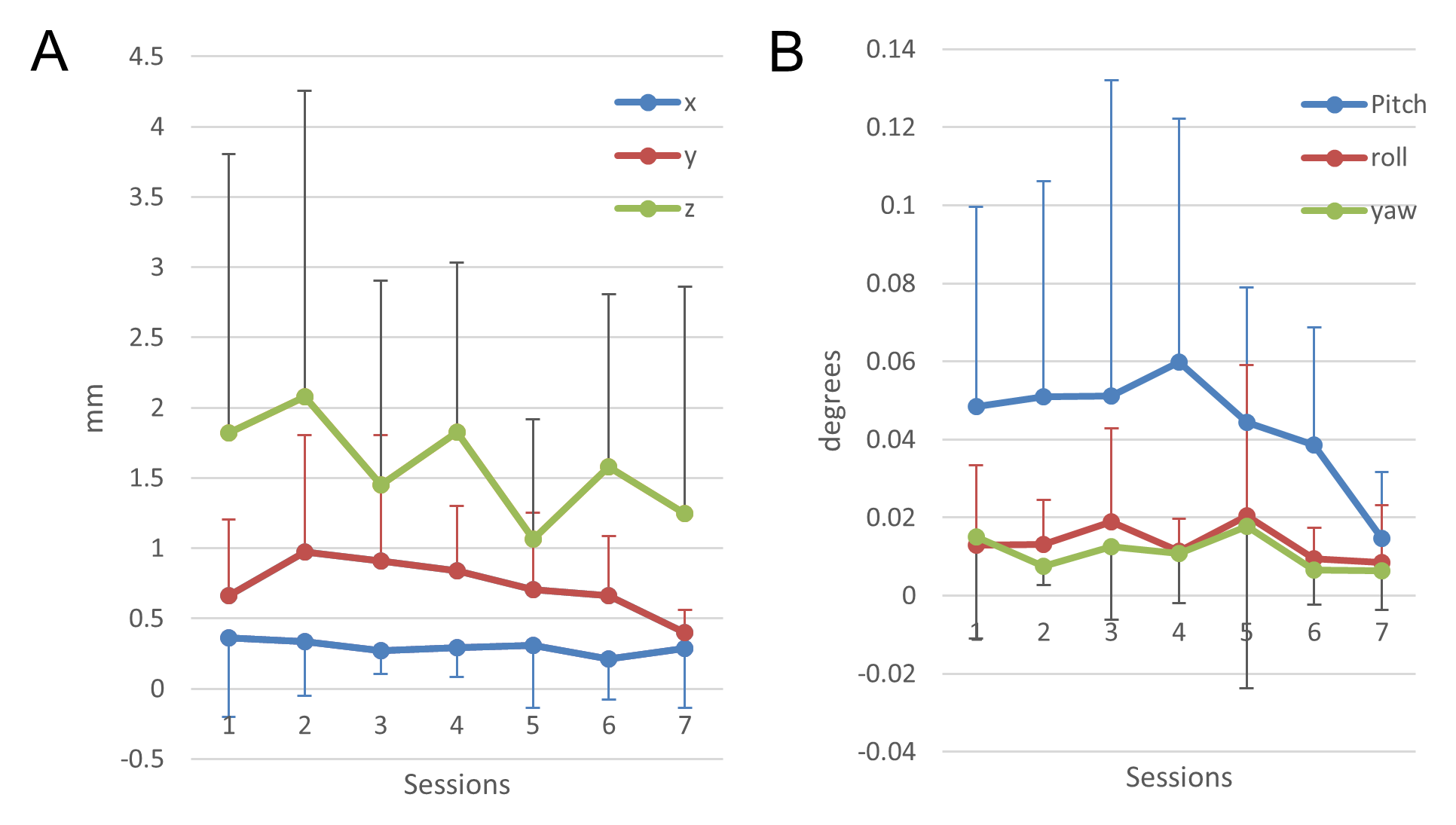

Supplement: S1 Fig — Average across participants of the maximal movement between volumes in each session. The error bars represent the standard deviation. (A) Translational movements. (B) Rotational movements. S5 Data. (TIF) [file pbio.2004103.s002.tif]

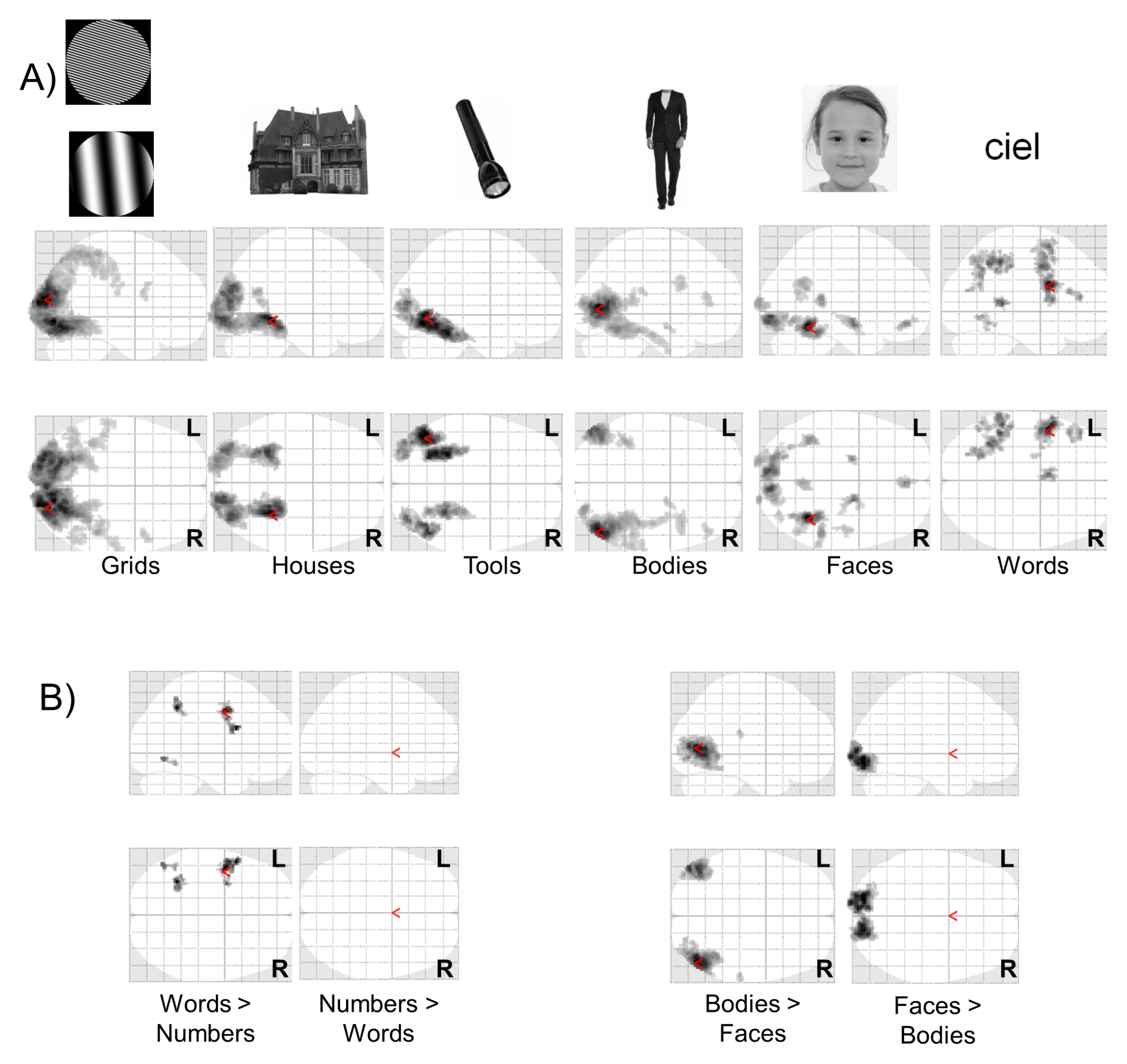

Supplement: S2 Fig — Each category was contrasted with the other pictures (omitting the grids). No significant cluster was observed for numbers. The red arrow indicates the global maximum for a given analysis. Bottom views (B) show additional contrasts of interest. L, left; R, right. (TIF) [file pbio.2004103.s003.tif]

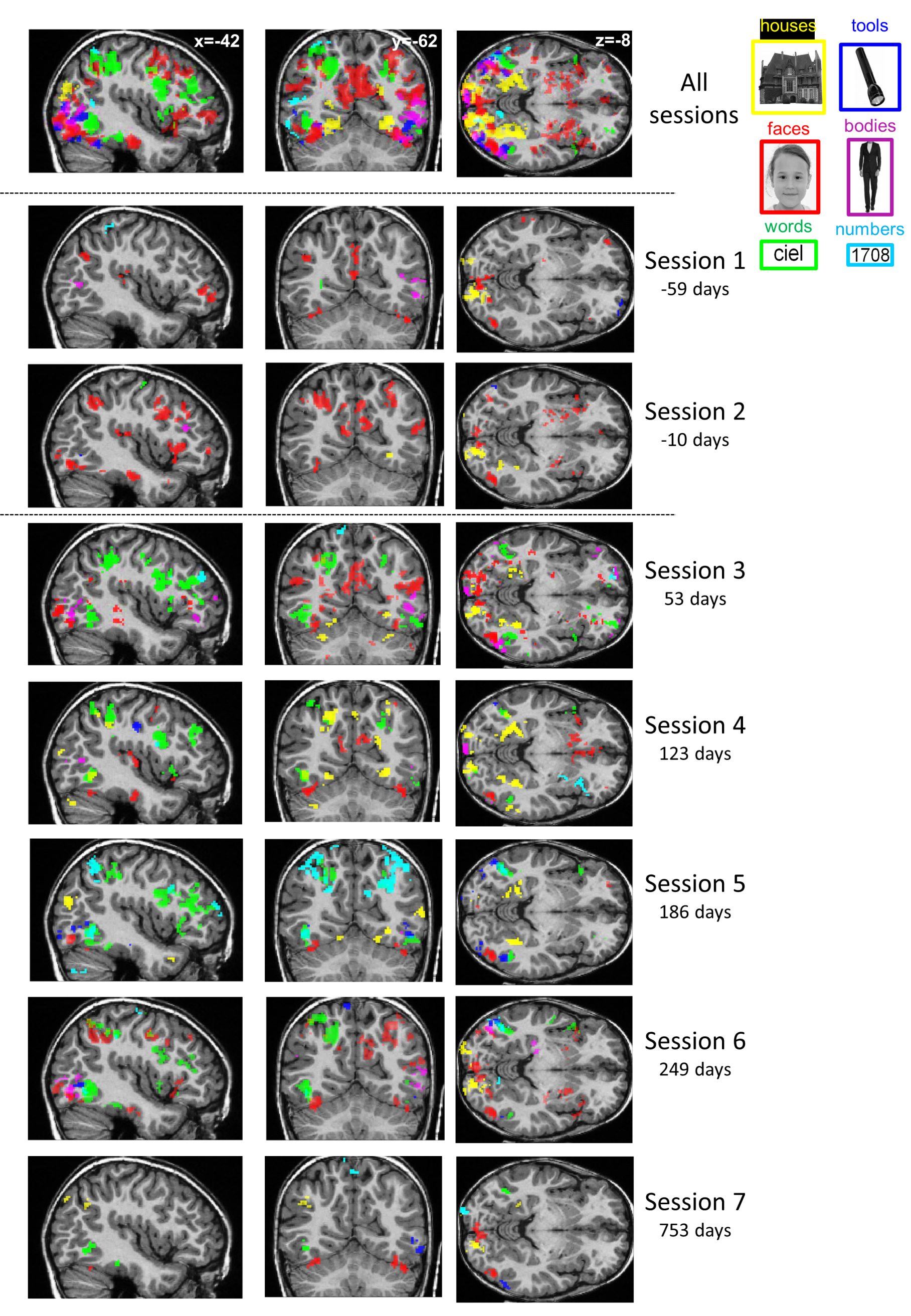

Supplement: S3 Fig — Each category is contrasted relative to the other pictures (omitting the grids), pooling across all 7 sessions in the first row and in each of the 7 sessions in the following rows (p < 0.001, FWE-corrected at the cluster level at p < .05). The scanning dates are indicated in days relative to the first day of school. The reading network (in green) is evident from session 3 (53 days after the onset of school) with a strong parietal and frontal component all along the first year of school (see also Fig 3). Note the response to numbers in session 5, which superposes to words in the left fusiform and bilateral parietal regions. In this child, activations to faces were clear and stable in the ventral areas, whereas activations to houses, tools, and bodies were less visible in each session—but the mosaic of category-specific regions is clearly organized when the statistical power is increased by pooling all sessions together (top row). FWE, family-wise error. (TIF) [file pbio.2004103.s004.tif]

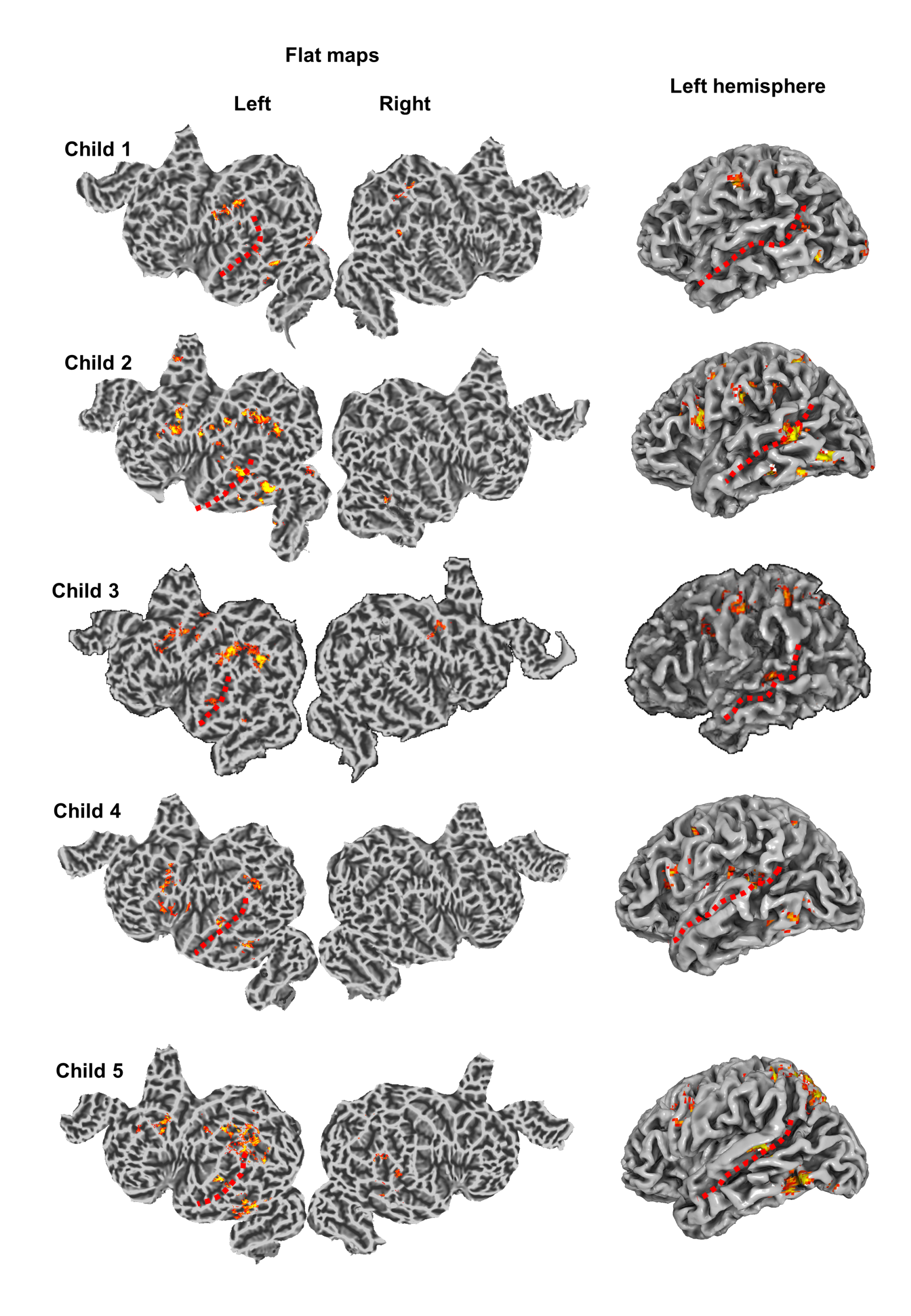

Supplement: S4 Fig — Children 1 to 5. The children are ordered from the best to the worst reader as determined by their LUM score at the last session (voxelwise p < 0.001 and clusterwise p < 0.05, FWE corrected). The red dotted line is placed along the left superior temporal sulcus. FWE, family-wise error; LUM, “Lecture en une minute.” (TIF) [file pbio.2004103.s005.tif]

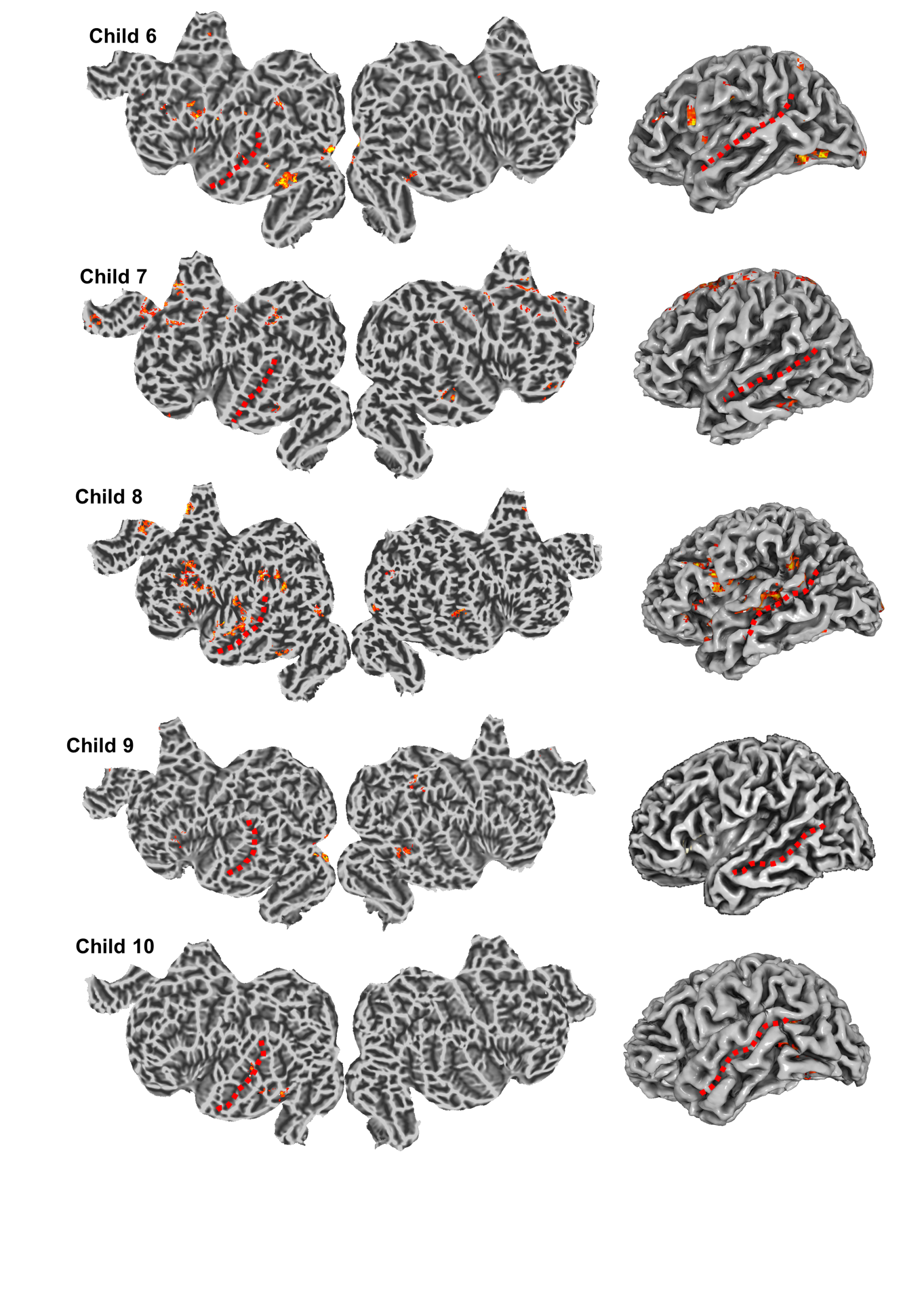

Supplement: S5 Fig — Children 6 to 10. (TIF) [file pbio.2004103.s006.tif]

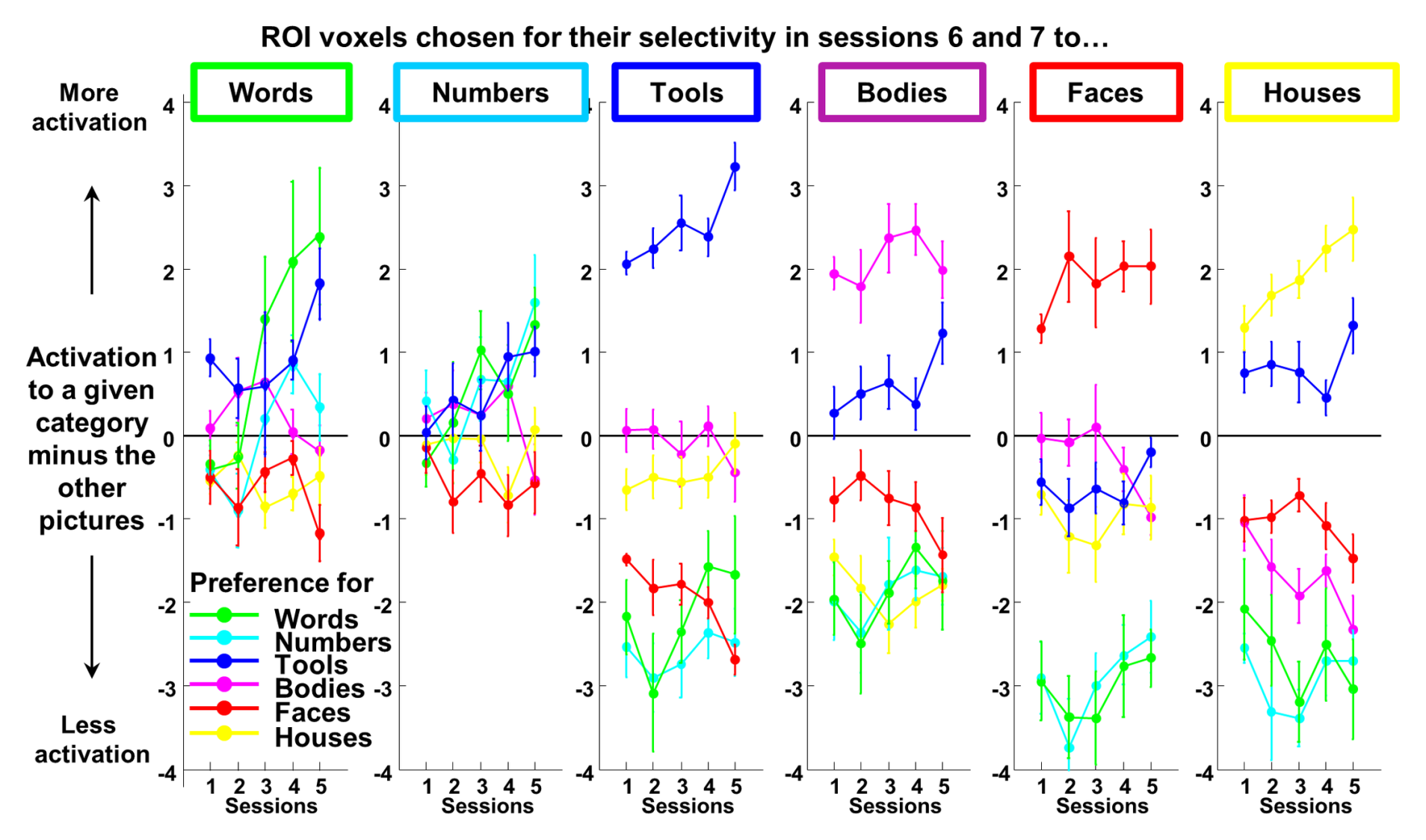

Supplement: S6 Fig — Same as Fig 6, except that the value being plotted is the contrast of one category minus the other picture stimuli (i.e., houses, faces, bodies, and tools), within the specific ROI determined in sessions 6 and 7 for the visual category indicated on the top of each panel. S3 Data. ROI, region of interest. (TIF) [file pbio.2004103.s007.tif]

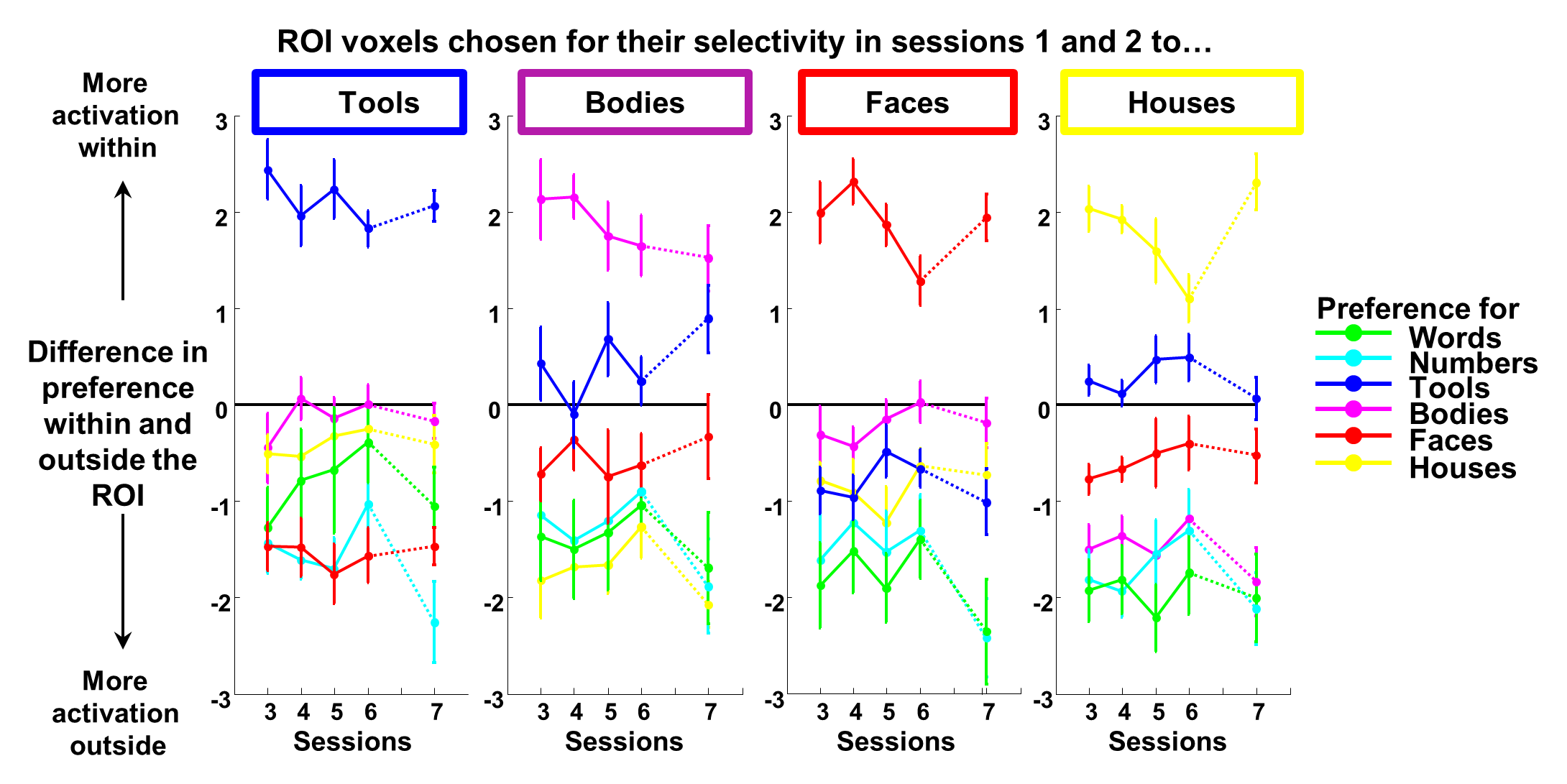

Supplement: S7 Fig — For each child, we identified the left ventral temporal voxels that were selective for a given category during session 1 and 2 (prior to schooling). At this age, no specialization was observed for words and numbers. The curves show how the responses of these voxels to the preferred and nonpreferred categories evolved in subsequent sessions 3 through 7 (same format as Fig 6). S6 Data. (TIF) [file pbio.2004103.s008.tif]

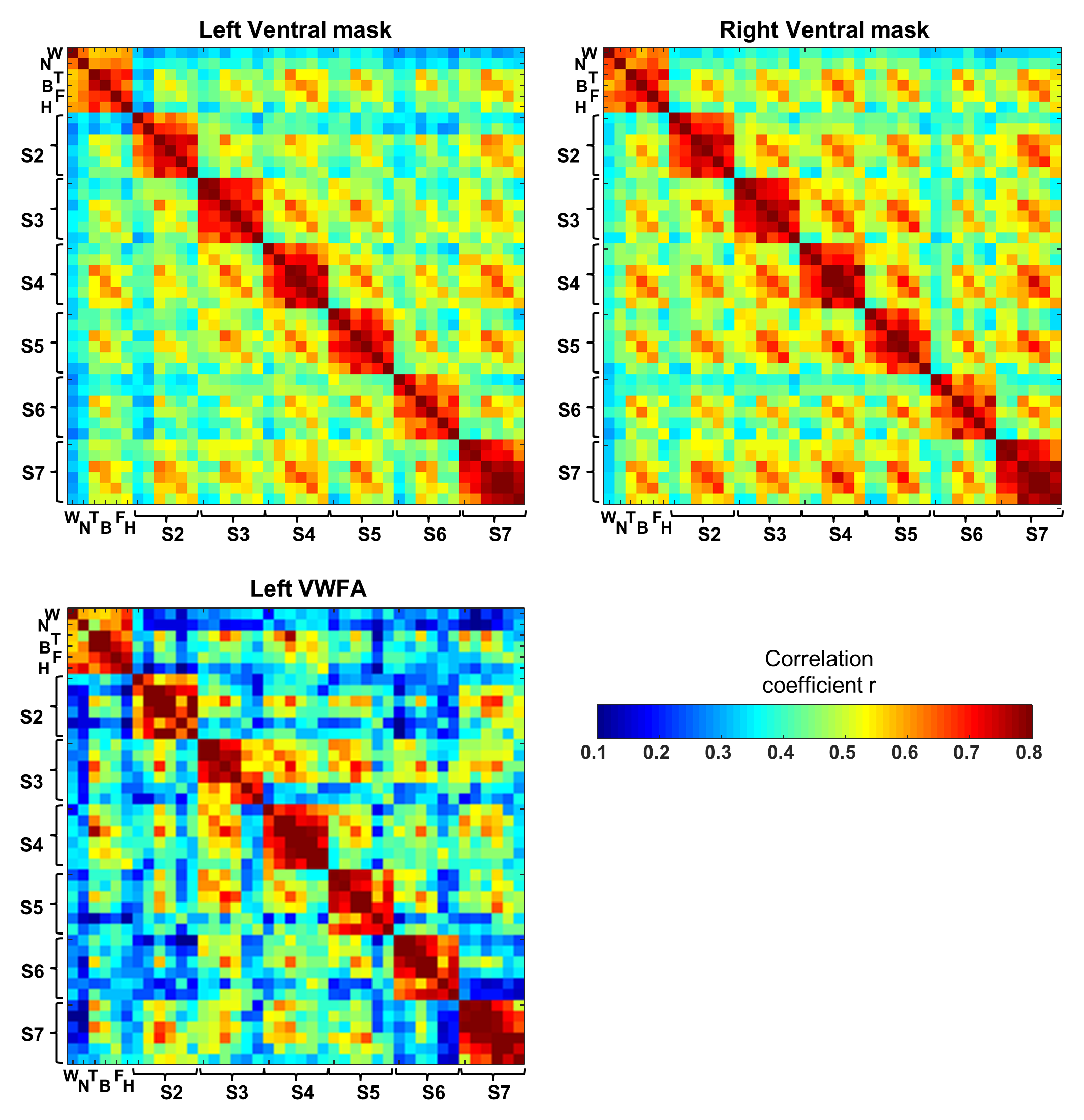

Supplement: S8 Fig — The t value of each contrast for one category versus all others was recovered in each voxel of the mask in each infant and each session. These images were then treated as a vector over voxels, and the correlations of these vectors were computed and averaged across subjects. Several effects can be seen: (1) a square pattern along the main diagonal shows a high similarity within each session; (2) diagonals in the adjacent squares demonstrate that the maps of activations are remarkably stable between one session and the next; (3) reproducible reddish squares illustrate that some categories induce similar activation patterns, e.g., bodies and tools in the left ventral mask or bodies, tools, and faces in the right ventral mask; (4) the activations evoked by words and numbers in sessions 1 and 2 are not correlated with those in the following sessions (2 blue lines at the top of the matrix). Stable patterns emerge after session 3. The activation pattern is roughly similar in the subject-specific VWFA (mask of the contrast words > others in sessions 6 and 7, voxel p < .001). B, bodies; F, faces; H, houses; N, numbers; S2–S7, sessions 2 to 7; T, tools; VWFA, visual word form area; W, words. (TIF) [file pbio.2004103.s009.tif]
